# A reassessment of risk associated with dietary intake of ochratoxin A based on a lifetime exposure model

**DOI:** 10.3109/10408444.2011.636342

**Published:** 2012-01-25

**Authors:** Lois A Haighton, Barry S Lynch, Bernadene A Magnuson, Earle R Nestmann

**Affiliations:** Cantox Health Sciences International, An Intertek Company

**Keywords:** Ochratoxin A (OTA), risk assessment, mechanisms, nephropathy, renal tumors, margin of exposure, threshold, genotoxicity, maximum limits

## Abstract

Mycotoxins, such as ochratoxin A (OTA), can occur from fungal growth on foods. OTA is considered a possible risk factor for adverse renal effects in humans based on renal tumors in male rats. For risk mitigation. Health Canada proposed maximum limits (MLs) for OTA based largely on a comparative risk assessment conducted by Health Canada (Kuiper-Goodman et al., 2010), in which analytical data of OTA in foods were used to determine the possible impact adopting MLs may have on OTA risks. The EU MLs were used for comparison and resultant risk was determined based on age–sex strata groups. These data were reevaluated here to determine comparative risk on a lifetime basis instead of age strata. Also, as there is scientific disagreement over the mechanism of OTA-induced renal tumors, mechanistic data were revisited. On a lifetime basis, risks associated with dietary exposure were found to be negligible, even without MLs, with dietary exposures to OTA three to four orders of magnitude below the pivotal animal LOAEL and the TD_05_. Our review of the mechanistic data supported a threshold-based mechanism as the most plausible. In particular, OTA was negative in genotoxicity assays with the highest specificity and levels of DNA adducts were very low and not typical of genotoxic carcinogens. In conclusion, OTA exposures from Canadian foods do not present a significant cancer risk.

## Contents

Abstract..........147
Introduction..........148
  Proposed OTA standards in Canada..........148
  Hazard assessment..........149
  Risks of human health effects associated with OTA..........150
  Mechanisms of toxicological effects in animals..........151
Genotoxicity of OTA..........152
DNA adduct formation..........154
Nongenotoxic modes of action..........155
  Induction of oxidative stress..........156
  Additional epigenetic mechanisms..........157
  Dose response assessment..........157
  Exposure assessment..........158
  Risk characterization..........160
  Derivation of lifetime MoE for OTA..........162
Summary..........164
Acknowledgments..........165
Declaration of interest..........165
References..........165

## Introduction

Mycotoxins, fungal metabolites resulting from fungal growth during food production and storage, can occur as natural contaminants in many foods, including those produced in or imported into Canada. Ochratoxin A (OTA) is one such mycotoxin of potential concern relating to the documented association of OTA with rodent renal tumors, in particular among male rats, and with organ toxicity in pigs. Food commodities that are susceptible to OTA contamination include wheat, oats, and rice, and other food products such as grapes, raisins, wine, corn, soy, coffee, and beer. Humans who typically consume these foods (essentially all consumers) would be expected to have some exposure to OTA. Given its natural origin, pervasiveness at low levels and chemical stability, it is not possible to entirely eradicate OTA from the diet.

For contaminants that may pose a health concern, risk-assessment practices may be used to determine a standard based on a dose or exposure that should be without appreciable risk to consumers. Risk assessment is a multistep process in which an exposure assessment and hazard assessment are typically conducted independently, and the results of these assessments are compared to provide an estimation of risk. In assessing exposures to contaminants from the diet, a quantitative estimate of dietary intake can be determined from concentration data of the contaminants in foods coupled with food intake survey data. The hazard assessment involves the evaluation of toxicology data which may be obtained from animal and/or human studies to define the inherent toxicity of a substance. Determining the dose-response curve of a contaminant is a component of the hazard assessment and the results can be used to derive a level of dietary intake that should not be associated with adverse effects in humans. The dose-response curve should be determined for the most sensitive adverse effect that is considered to be mechanistically relevant to humans. The results of the exposure assessment can be compared to this dose to characterize the probability of adverse effects.

OTA and other mycotoxins are just one category of contaminants that may commonly be found in foods. Other chemical contaminants that may be found in foods and/or drinking water include heavy metals (e.g. lead, mercury, arsenic), residual pesticides, animal drug residues, and persistent environmental contaminants such as dioxins and polychlorinated biphenyls. Further to these are food-processing agent residues, food contact material migrants, contaminants formed during the cooking of food, indirect food additives, as well as a wide array of naturally occurring mutagens and carcinogens that are normal constituents of foods. For some of these contaminants, chemical-specific maximum concentrations or residue limits have been established. These may be adopted as a voluntary or regulatory standard for specific food commodities or products. Deriving a maximum limit or standard may be based on health concerns or, if the ability to reduce the contaminant is diminished by technological limitations, principles of “as low as reasonably achievable/practicable” (ALARA or ALARP) may be adhered to instead. In most other situations, the implementation of Good Agricultural Practices (GAP) and Good Manufacturing Practices (GMP) are counted on to ensure that food products are of high quality and do not present a health risk.

However, stringent standards implemented for contaminants such as mycotoxins that can be controlled through GAP and GMP could result in a greater frequency of noncompliance and possible food product detentions and recalls, but may have little impact on reducing health risks. Wu (2004) conducted a risk assessment and analysis of the economic impacts versus health benefits that may be expected from tighter global regulation of fumonisins and aflatoxins. The author's findings were that economic losses from the adoption of more stringent standards for these mycotoxins than those of the United States for agricultural producers would be in excess of 100 million dollars and would be unlikely to improve health significantly.

In contrast, adopting standards to prevent unlawful practices, such as adding melamine to infant formula to show a falsely higher protein content, are of benefit to protecting health. Therefore, in deriving standards, the ultimate aim should be to achieve optimal voluntary or regulatory maximum limits for human safety, which at the same time do not become technical barriers to trade or impose costs to food producers and consumers that are not outweighed by positive health outcomes.

The primary purpose of this paper is to reevaluate the data published in the Health Canada risk assessment (Kuiper-Goodman et al., 2010) and assess risk on a lifetime basis rather than individual age strata. This review was accomplished by evaluating the results of exposure assessments for OTA in food using data from Canada and by comparing these exposures to doses of OTA anticipated to be without appreciable risk. Furthermore, given the disagreement among different regulatory agencies on the mechanisms of carcinogenic activity of OTA, a critical review of the genotoxic potential of OTA was undertaken. It is well understood that OTA is very stable and resilient to primary and further food processing such as grain milling and baking. Grain milling results in a redistribution of OTA among the milled grain fractions such that some fractions exhibit lower OTA levels whereas others exhibit higher levels than present in the unprocessed grain. We have therefore also reviewed the assumptions regarding processing factors that were incorporated into the risk assessment.

A secondary objective is to review the need for, and assumed efficacy of, maximum limits for OTA in selected foods that are currently proposed by Health Canada, based on our reevaluation of the Health Canada risk assessment.

### Proposed OTA standards in Canada

Standards for OTA have been adopted by the European Union (EU) and proposed by the government of Canada. The United States does not currently have standards for OTA. Proposed standards for Canada and the existing European Union standards are summarized in Table 1. Although Canada has not proposed standards for all of the same food groups for which there are EU standards, the proposed maximum limits are very similar.

**Table 1:** Standards for ochratoxin A.

Food commodity	European Union^a^ maximum levels (μg/kg)	Canada^b^ (proposed) maximum limits (ppb)
Unprocessed cereals/raw cereal grains^c^	5	5
All products derived from unprocessed cereals, including processed cereal products and cereals intended for direct human consumption with exception of foods for infants and young children and foods for special dietary purposes	3	
Direct consumer grains (i.e., rice, oats, pearled barley)		3
Derived cereal products (flour^d^)		3
Derived cereal products (wheat bran)		7
Breakfast cereals		3
Grape juice (and as ingredients in other beverages) and related products (concentrated grape juice, grape nectar, grape must intended for human consumption)	2	2
Dried vine fruit (currants, raisins, sultanas)	10	10
Wine, fruit wine, aromatized wine, aromatized wine-based drinks and aromatized wine-product cocktails	2	
Roasted coffee beans and ground roasted coffee excluding soluble coffee	5	
Soluble coffee (instant coffee)	10	
Baby foods and processed cereal-based foods for infants and young children	0.5	0.5
Dietary foods for special medicinal purposes intended for infants	0.5	0.5

a Commission Regulation (EC) No 1881/2006 of 19 December 2006 (also includes spices and liquorice; Commission of the European Communities, 2006);

b Health Canada's proposed Maximum Limits (Standards) for the Presence oftheMycotoxin Ochratoxin A in Foods in February 2009 (Health Canada, 2009)';

c takes into consideration the reducing effect of processing or redistribution;

d For bread, pastries and other flour-based foods, the guidelines to pertain to the flour portion. In the future, based on further monitoring data, Health Canada may consider modifying these maximum limits (MLs), or introduce MLs for products not yet covered.

The EU standards were adopted based on the 2006 opinion of the Scientific Panel on Contaminants in Food (EFSA, 2006) and the tolerable weekly intake (TWI) of 120 ng/kg body weight. The results of an assessment of dietary intake of OTA by the population of EU Member States conducted by Istituto Superiore di Sanitá-Rome-Italy was used to select numerical values for the maximum levels for the specific food commodities (EFSA, 2006). The Canadian standards appear to be based in part on the EU standards and in part on the results of a Risk Assessment of OTA for all Age-sex Strata in a Market Economy completed by Health Canada (Kuiper-Goodman et al., 2010).

### Hazard assessment

Toxicities of OTA demonstrated in animal studies include nephrotoxicity, teratogenicity, carcinogenicity, and immunosuppression; however, species and gender-related differences in sensitivity have been noted. Detailed discussions of OTA toxicity studies are provided elsewhere (Kuiper-Goodman and Scott, 1989; EFSA, 2006; JECFA, 2008; Ringot and Chango, 2010). In a recent review, differences in the metabolism of OTA by various species is discussed as a factor contributing to species differences in sensitivity to OTA toxicity (Wu et al., 2011). OTA is subject to metabolism by several different pathways including hydrolysis, hydroxylation, lactone-opening, and conjugation, yielding numerous different metabolites. The major metabolite in animals and humans is ochratoxin-alpha which is readily excreted and considered to be nontoxic (Wu et al., 2011). Several other metabolites of varying toxic potential have been identified in urine or feces of rats, both of which are important routes of excretion as OTA is metabolized by the kidneys, liver, and intestines. The rat is thus far the only species in which a lactone-opened form of OTA with higher toxicity as compared to OTA, has been identified. The major metabolite in pigs from the hydroxylation pathway is 4-(S)-hydroxyochratoxin A, whereas 4-(R)-hydroxyochratoxin A is the primary hydroxyl metabolite in rats and humans. Another metabolite identified as 10-hydroxyochratoxin A has been found only in rabbits. Further study is needed to characterize the toxicity of the various metabolites and to clarify if differences in sensitivities of animals to OTA is related predominantly to different metabolic profiles.

The lowest dose effect noted in animal studies was renal function changes in pigs, which occurred at a dose of 8 μg/kg body weight/day. The lowest observed-effect level of 8 μg/kg body weight/day was determined from a 90-day subchronic study, in groups of 6 to 11 pigs, in which renal enzyme changes, and changes in renal function were noted at all doses (Krogh et al., 1974). In a follow-up study (Krogh et al., 1979), exposure of six pigs to approximately 40 μg/kg body weight/day for 2 years led to progressive nephropathy but not renal failure. One striking difference observed in the kinetics of OTA in the pig, which may explain this species greater sensitivity to OTA is provided by work of Hagelberg et al. (1989) who calculated clearance of OTA by renal filtration using glomerular filtration rates and concentrations of free fractions of OTA in plasma. Most species (quail, mouse, rat, monkey, and man) have a calculated clearance in the range of 1 to 14%, which are all at least 10 fold lower than the 110% value in the pig (Hagelberg et al., 1989). Other species differences in toxicokinetics of OTA have been recently reviewed (Coronel et al., 2010).

At higher doses, 50 μg/kg/d, OTA also caused an increased incidence of kidney tumors in rat studies, which was particularly evident in male rats (National Toxicology Program [NTP], 1989). The renal tumor results of the NTP study are provided in Table 2. The tumorigenic response occurred at doses higher than those associated with effects in pigs, however cancer is considered a more sensitive endpoint if associated with a nonthreshold mechanism of action. The likely mechanism for renal tumors in rats was evaluated and the results are discussed below.

**Table 2:** Numbers of rats with renal tumors in the 2-year gavage studies of ochratoxin A.

	Male				Female			
		
Site/Lesion	Vehicle control	21 μg/kg (adjusted dose 15 μg/kg/d)	70 μg/kg(adjusted dose 50 μg/kg/d)	210 μg/kg (adjusted dose 150 μg/kg/d)	Vehicle control	21 μg/kg (adjusted dose 15 μg/kg/d)	70 μg/kg (adjusted dose 50 μg/kg/d)	210 μg/kg (adjusted dose 150 μg/kg/d)
Number examined	50	51	51	50	50	51	50	50
Kidney tubule								
Adenoma, solitary	1	1	5	10	0	0	1	3
Adenoma, multiple	0	0	1	0	0	0	0	2
Carcinoma, solitary	0	0	12	20	0	0	1	3
Carcinoma, bilateral/multiple	0	0	4	10	0	0	0	0
Renal tubule cell adenomas and carcinoma combined	1	1	22	40	0	0	2	8
Metastatic renal carcinoma (all sites)	0	0	4	13	0	0	1	0

Source: Summarized from results reported in the NTP (1989) rat study.

### Risks of human health effects associated with OTA

In contrast to the animal data, evidence from human studies to demonstrate adverse effects of OTA at current dietary exposures, is inadequate. Based on rodent and pig studies, it is reasonable to assume that OTA, at high-enough exposures, would cause similar toxicities in humans. However, no epidemiology studies have been identified that conclusively link OTA from the diet to adverse health effects in humans. Although several studies have implied that OTA may be responsible for a human condition called Balkan Endemic Nephropathy (BEN), and OTA was reported to be present in crops in higher concentrations in BEN-endemic areas compared to non-endemic areas (Gluhovschi et al., 2011), this association has not been proven, and recent research into BEN is increasingly implicating aristolochic acid as the etiological agent. Aristolochic acids were just recently listed in the Twelfth Report on Carcinogens as substances known to be human carcinogens (NTP, 2011). Moreover, the recent review on biomarkers of OTA exposure highlights the challenges of monitoring OTA exposure in humans, and the lack of correlation between dietary exposure and blood levels (Duarte et al., 2011).

BEN is a chronic degenerative kidney disease associated with a high incidence of upper urothelial cancer in humans. Upper urothelial cancer, which includes tumors of the ureter and bladder lining, is not the same tumor as noted in rat studies in which the renal tubule cells were the target cell type at high doses, but it is one of the primary tumor types observed in humans that have consumed supplements containing aristolochic acids, and in animal studies with aristolochic acids (NTP, 2011). It is noted that the inclusion of aristolochic acids in the Report on Carcinogens occurred after the Kuiper-Goodman et al. (2010) risk assessment was published.

The endemic nature of BEN suggests that some humans have a genetic predisposition to developing the disease, which makes them susceptible to an etiological agent that is likely to be an environmental factor. Environmental factors that have been suggested as the possible etiological agents include lead, cadmium, polycyclic aromatic hydrocarbons, selenium deficiency, viruses, OTA, and aristolochic acid (Mally et al., 2007). Of these suggested agents, OTA and aristolochic acid were the most plausible. However, more recent research favors aristolochic acid as the causative agent (Nedelko et al., 2009; Slade et al., 2009; Stefanovic and Polenakovic, 2009; Stefanovic et al., 2009). Earlier publications also proposed that aristolochic acid and not OTA may be the causative agent in BEN (Cosyns et al., 1994; Arlt et al., 2007; Grollman et al., 2007).

Aristolochic acid is the causative agent in Chinese herb nephropathy (CHN) which has been renamed aristolochic acid nephropathy (AAN). The morphological and clinical profiles of BEN and aristolochic acid nephropathy are similar (Stefanovic and Polenakovic, 2009). In contrast, the histological origin of the tumors associated with BEN (upper urothelial cancer) is not the same as that of the tumors observed in the OTA rat studies (renal tubule—outer strip of outer medulla; Mally et al., 2007). Also, OTA and aristolochic acid differentially alter the regulation of expression of vascular endothelial growth factor (VEGF) in porcine kidney epithelial cells, as well as activity of the transcription factors that regulate VEGF (Stachurska et al., 2011).

The source of exposure to aristolochic acid was suggested to be wheat inadvertently contaminated with seeds from the plant *Aristolochia clematitis* (Grollman and Jelakovic, 2007). A mechanism explaining the higher incidence of BEN-associated cancers in the Balkan region with aristolochic acid as the causative agent was proposed by Nedelko et al. (2009). Following the discovery of adenosine (A) to thymine (T) mutations in the *p53* tumor suppressor gene of tumor DNA samples from BEN patients (Grollman et al., 2007), further studies of aristolochic acid were conducted which demonstrated that aristolochic acid induced these A to T mutations in human *p53* sequences *in vitro.* These particular *p53* mutation sites, which are rarely mutated in human cancers in general, are found in BEN patients (Nedelko et al., 2009). Slade et al. (2009) also reported finding aristolochic-DNA adducts and the A to T transversion *p53* mutations in the tumor tissue from patients with BEN in Croatia and Bosnia. A literature search neither found *in vitro* studies which reported OTA induced A to T transversions nor other mutagenic responses in human *p53;* however, activation of *p53* was reported to inhibit OTA-induced apoptosis in monkey and human kidney epithelial cells (Li et al., 2011).

OTA is less likely than aristolochic acid to induce A to T transversions in the *p53* gene of BEN patients as it is a weak mutagen at best (Nedelko et al., 2009), and the results of another study have shown that hypoxanthine-guanine phosphoribosyl transferase (HPRT) gene base substitutions of mammalian cells exposed to OTA did not include A to T transversions, and were similar to the spontaneous mutation pattern (Palma et al., 2007). Following an international symposium in Zagreb, Croatia on BEN that was held in October 2006, Grollman and Jelakovic (2007) reported that the cardinal conclusion emerging from the meeting was that aristolochic acid, and not OTA, was the primary risk factor for BEN and associated upper urothelial cancer. However, clarification of the slower progression to end-stage renal disease, and longer cumulative period for the occurrence of urothelial cancer for BEN in comparison to AAN is still needed (Pfohl-Leszkowicz, 2009; Slade et al., 2009). Thus, although both OTA and aristolochic acid have been demonstrated to nephrotoxic in animals, the specific pathology of BEN and associated urothelial cancers in humans has been demonstrated to be more closely reflective of that induced by aristolochic acids.

## Mechanisms of toxicological effects in animals

The mechanism by which OTA induces renal tumors in rats is uncertain. In the absence of definitive data, the approach for assessing the potential health hazards of OTA to humans differs among some regulatory agencies. Health Canada has taken the position that the mechanism is nonthreshold, a default position based largely on a finding of equivocal genotoxic potential (Kuiper-Goodman et al., 2010). The European Food Safety Authority (EFSA) concluded that the mechanism for carcinogenic response of OTA was most likely threshold based, the significance being that low doses below the threshold dose would not be associated with a cancer risk (EFSA, 2006).

The lowest dose of OTA administered to male and female F344 rats in the cancer bioassays (21 μg/kg body/ weight, administered by gavage 5 days per week) was not associated with an increased incidence of renal tumors (Table 2). The incidence of renal tumors was also very low in the female mid-dose group. These data support a threshold-based mechanism.

Furthermore, a recent publication (Mantle and Nagy, 2008) demonstrating binding of OTA to α-2u-globulin, a classical species- and gender-specific response of male rats, which may, at least partially, explain the uncommonly potent response of OTA in male rats in the NTP gavage study. This study, which included only a few animals and can be considered as a hypothesis generating study, nevertheless contributes to the data supporting a threshold-based mechanism of action.

Using exponential dose-response relationship modeling, the tumor data from the F344 rats in the NTP study and data from a study in Dark Agouti male rats fed OTA for 2 years (Mantle et al., 2005) indicate that OTA is a threshold carcinogen (Mantle and Nagy, 2008).

In recent studies with male Dark Agouti rats (20/group), the incidence of renal tumors was not increased following administration of 400 ppb OTA in the diet (∼50 μg/kg body weight/day decreasing to 20 to 30 μg/kg body weight/day for adults) from 8 weeks of age to 2 years (Mantle, 2009). Three additional groups (20/group) were administered 5 ppm OTA in the diet for various periods of time (3 months, 6 months, and 9 months) after which OTA was discontinued and the animals observed over the course of their natural life. Three months of continuous exposure to 5 ppm OTA (doses ranging from 640 μg/kg/day at study commencement and declining to 450 μg/kg at 3 months, according to growth) was not associated with an increased incidence of renal tumors or other obvious pathological changes at the end of the observation study period. In the groups of male rats administered 5 ppm OTA for 6 months, and 9 months, 1 out of 20, and 4 out 20 rats, respectively, developed renal tumors before the end of the study. However, it should be noted that the number of animals per group was less than the number used in the NTP bioassay. Also, this study may have benefited by the inclusion of a lifetime treatment group for comparative purposes.

Mantle (2009) plotted the renal tumor incidence data from all recent rat studies with accumulative dose on a logarithmic scale, as per the approach of Waddell (2006). Similar to the data for the NTP rat study evaluated by Waddell (2006), a threshold for OTA was demonstrated for all strains. In addition to the NTP rat data, Mantle (2009) included results from studies in Fischer, Fischer/ Sprague-Dawley cross, Dark Agouti, and Lewis rats. Mantle (2009) concluded that the dietary studies demonstrated a two fold higher threshold for carcinogenic effects, in comparison to that observed for the gavage NTP (1989) rat study. In addition, Mantle (2009) noted that under the 3-month exposure protocol followed by an exposure-free period for the rest of the animals' lifetime, exceptionally high doses of OTA (i.e., 450 to 650 μg/ kg/day) failed to elicit any evidence of a nephrocarcinogenic effect in Dark Agouti rats. Likewise, Mantle and Kulinskaya (2010), in a study similar in design to Mantle et al. (2005), reported that administration of OTA in the diet of F344 rats at a dose of 50 μg/kg/day for 2 years resulted in the formation of unilateral renal carcinomas in 4 rats out of 34 included in the test group. Mantle and Kulinskaya (2010) concluded that the gavage mode of dosing in the NTP (1989) study likely optimized conditions for tumor development, whereas dietary exposure is of more relevance to the human situation. Vettorazzi et al. (2011) recently reported that administration of OTA to fasted versus fed rats resulted in increased bioavailability of OTA, and higher maximum observed concentrations in both kidney and liver, with adult male rats showing the greatest difference due to fasting. It is noted that these latter two studies were not published until after the Kuiper-Goodman et al. (2010) study.

The significantly higher kidney tumor incidence following OTA exposure has been observed in males versus females in both rats and mice. Proposed mechanisms for the gender difference in susceptibility have included differences in OTA transporters in the kidney, differences in expression of cytochrome p450 enzymes involved in the metabolism of OTA, and the presence of α-2u-globulin in the adult male rat. Although a role for α-2u-globulin in the dramatic difference between male and female rat kidney tumor incidence was originally dismissed, recent studies have reevaluated this male rat-specific mechanism. Studies by Mantle and Nagy (2008) demonstrating OTA binding to α-2u-globulin, specific rapid delivery of OTA to the proximal tubule epithelium by the α-2u-globulin protein, and reexamination of the renal tumor histology from the NTP study support a contributory role of this protein in the high incidence of renal tumors in male rats (Mantle and Nagy, 2008). The α-2u-globulin, which binds to OTA, is an androgen-dependent rat-specific protein. In addition to the NTP study with F344/N rats, male-specific susceptibility to OTA-induced renal tumors has been observed in Dark Agouti rats (95% males with renal tumors compared to 3% females) and in Lewis rats (80% males; 25% females; Castegnaro et al., 1998).

The binding of OTA to α-2u-globulin results in rapid delivery of OTA to the kidney proximal tubule epithelia by α-2u-globulin in males, creating a much higher concentration of OTA at the target site, with greater toxicity and nephropathy than would occur in females. The binding and removal of OTA by α-2u-globulin in male rats also results in a shorter elimination half-life for OTA than in female rats. Higher plasma OTA levels after dosing have been observed in female compared to male rats of various strains including the Fischer/Sprague Dawley cross (Mantle, 2009), Fischer (Zepnik et al., 2003), and Dark Agouti (Castegnaro et al., 2006). One explanation for this difference is that in males, binding of OTA to α-2u-globulin and rapid delivery of OTA to the kidney, increases the rate of elimination from the blood, lowering the OTA levels in blood. Thus, although the females have higher blood concentrations and a longer half-life of OTA, they are less susceptible to OTA renal carcinogenesis than males (Dietrich et al., 2005) because of a lower concentration at the target site. The lower tumor incidence seen in male rats in response to continuous dietary OTA as compared to intermittent oral gavage, despite higher doses given in the diet studies, may also be attributed to the fact that the gavage protocol would result in surges of OTA delivered to the tubule as compared to lower but more constant levels presented from feed intake (Dietrich et al., 2005).

There appears to be no human equivalent of the male rat urinary globulins (Mantle and Nagy, 2008). Therefore, these authors have stated that extrapolation from dose-response data from experimental rat renal carcinoma for human risk assessment may be appropriate only for female rat data.

It is proposed that the rapid delivery of OTA to the kidney proximal tubule epithelium would result in a concentrated surge of OTA, resulting in higher toxicity and nephropathy than would occur with slower release of OTA from plasma proteins in females or from OTA in the diet. Repeated rounds of high concentration surges of OTA and subsequent cell damage, repair, and compensatory cell proliferation are proposed as the mechanism of tumor formation (O'Brien et al., 2001; Dietrich et al., 2005; Mantle, 2009). This mechanism can explain the reduced tumor formation in females as compared to male rats, fewer tumors resulting from dietary exposure as compared to gavage despite higher doses delivered by diet (Mantle, 2009), and the lack of subsequent tumor formation in rats fed high doses of OTA for short periods (Mantle, 2009). These observations support a threshold mechanism for carcinogenicity of OTA (threshold corresponds to a dose of about 16,700 ng/kg body weight/day).

## Genotoxicity of OTA

Conclusions regarding the genotoxicity of OTA, which would help to address the question of mechanism, also are ambiguous.

There exists an extensive body of historical data with respect to the genetic toxicity of OTA. These data have been reviewed by the International Agency for Research on Cancer (IARC, 1993), the Joint FAO/WHO Expert Committee on Food Additives (JECFA, 2008), and others (Brambilla and Martelli, 2004) and briefly summarized below. In an earlier Health Canada risk assessment on OTA by Kuiper-Goodman and Scott (1989), it was concluded that the available data supported that OTA was not mutagenic, but that it was weakly genotoxic to mammalian cells based on single-strand breaks. It was noted that, although there was no general agreement on the significance of single-strand breaks, the authors did consider these changes as indicating genotoxicity, as they were concluded to be the result of a deleterious interaction of OTA with the hereditary material of the cell (Kuiper-Goodman and Scott, 1989).

In general, in the Ames assay, OTA has been shown to be inactive (i.e., negative), both with and without the presence of an exogenous source of metabolic activation (e.g., Wehner et al., 1978; Bartsch et al., 1980; Bendele et al., 1985; Zeiger et al., 1988; NTP, 1989; Wurgler et al., 1991; Zepnik et al., 2001; Föllman and Lucas, 2003).

The SOS spot test in *Escherichia coli,* when conducted at noncytotoxic concentrations, has also produced exclusively negative results with OTA (Auffray and Butibonnes, 1986; Krivobok et al., 1987; Malaveille et al., 1991). Similarly, in mammalian cell assays such as the HPRT assay in V79 hamster cells (Bendele et al., 1985) and the TK L5178Y mouse lymphoma assay (Föllman and Lucas, 2003), OTA has shown no evidence of mutagenic activity.

The results of *in vitro* studies that have assessed the potential for OTA to cause DNA single-strand breaks, or other cytogenetic effects in mammalian cell lines are mixed, with both positive (Mori et al., 1984; Creppy et al., 1985; Štětina and Votava, 1986; Manolova et al., 1990; Ehrlich et al., 2002; Ali et al., 2010) and negative results (Cooray, 1984; Bendele et al., 1985; Štětina and Votava, 1986; NTP, 1989) reported. Variable results have been reported in the comet assays using *in vitro* exposures (Ehrlich et al., 2002; Lebrun et al., 2006; Simaro-Doorten et al., 2006; Arbillaga et al., 2007; Klaric et al., 2010). Likewise, inconsistent results have been reported in non-standard *in vitro* cytogenetic assays (Föllman et al., 1995; Degen et al., 1997; Dopp et al., 1999).

As with the *in vitro* sister-chromatid exchange, DNA strand breakage, and cytogenetic assays, the available *in vivo* data are also difficult to interpret due to conflicting results or the use of high doses that cannot be extrapolated to the results of the carcinogenicity studies. For example, although positive results were reported for chromosome aberrations in the mouse at doses of 1 μg/ kg body weight/day in the diet for up to 45 days (Bose and Sinha, 1994; Kumari and Sinha, 1994), only a weak, nonstatistically significant response was reported in rats treated at up to 2,000 μg/kg body weight/day for 5 days (Mally et al., 2005a). No sister-chromatid exchanges were noted in response to gavage treatment of Chinese hamsters at OTA doses of 25 to 400 mg/kg body weight (Bendele et al., 1985).

Zeljezić et al. (2006) reported the results of a study in which OTA was assessed for capacity to induce DNA damage in the rat kidney in an *in vivo* alkaline comet assay. The authors administered OTA to groups of five adult female Wistar rats by intraperitoneal injection, dissolved in Tris buffer, at doses of 0.5 mg/kg body weight/ day for either 7, 14, or 21 days. Tissue analysis revealed the presence of OTA at mean concentrations of 4.86, 7.52, and 7.85 μg/mL in plasma after the 7-, 14-, and 21-day treatments, respectively. Corresponding concentrations in kidney tissue were 0.87, 0.99, and 1.09 μg/g respectively. At all treatment durations, the tail length, intensity, and tail moment were significantly increased relative to the controls. The highest tail was noted after 21 days of treatment. This study demonstrates genotoxic potential *in vivo,* following intraperitoneal OTA administration. The oral route of exposure and lower doses would have more closely mirrored potential human exposures. Also, it would have been useful to have tested male animals as the tumorigenic response in the kidney is much stronger in males.

Recently, Hibi et al. (2011) investigated the *in vivo* mutagenicity of OTA in the kidney tissue of F344/NSlc-Tg (*gpt*delta) transgenic rats. These rats carry ∼5 tandem copies of the transgene lambda EG10 per haploid genome. Point mutations in these reporter genes are identified by 6-thioguanine *(gpt* gene), whereas deletions in *red/ gam* genes are detected by Spi(-) selection. In an initial experiment, groups of four to five rats of each sex were treated with OTA by oral administration at a dietary dose of 5 ppm for either 4 or 13 weeks. Following treatment, the kidneys were harvested, a portion of which was used for histological assessment while the remainder was used for assessment of mutations in the reporter genes and measurement of 8-OHdG levels. Although exposure for up to 13 weeks reportedly induced karyomegaly and apoptosis in the outer stripe of the outer medulla, no effect on the mutation frequencies in the recovered *gpt* or *red/gam* genes was reported. A second experiment was conducted in males only that followed a similar protocol as the initial experiment, except for a 4-week only exposure and, instead of using whole kidney preparations, the kidney tissue was macroscopically separated into cortex and outer and inner medullae, prior to analysis. The mutation frequencies of the *gpt* gene in the cortex and inner and outer medullae were unaffected by OTA treatment. In contrast, the Spi^-^ mutation (deletion mutations) frequencies from the outer medulla were significantly greater (about three fold) than the controls. The authors considered this result of particular significance as the outer medulla contains the S3 segment of the proximal tubules, the site of tumor initiation. There were no changes to 8-OHdG levels in either experiment. Based on these results, the authors concluded that a genotoxic mode of action is likely, and that oxidative stress is not likely involved in OTA carcinogenesis. Although the study indicates that OTA is associated with deletion mutations in the target tissue, the potency of effect was low, especially in relation to effects reported in this assay for known genotoxic chemicals such as N-nitrosopyrrolidine and amino-3-methylimidazo[4,5-f]quinoline (Kanki et al., 2005) and in relation to the potency at which OTA induces kidney tumors in rats at very low doses. Essentially, although the data reported by Hibi et al. (2011) indicate some potential for genotoxic activity under the conditions of the assay, the role of this activity in the induction of kidney tumors is unclear.

Given the extensive testing of OTA in various genotoxicity batteries, including a number of nonstandard protocols and the use of high concentration and high-dose exposures, it is not surprising that the results of these studies are mixed. This may be partly due to chance and in some cases due to artifacts (i.e., product formed during processing or due to methodology; Brusick, 1986; Galloway et al., 1991), differences in experimental protocols and cell lines, use of high versus low concentrations and doses, or improper interpretation of the data (Green and Muriel, 1976). Although genotoxic activity was observed under some assay conditions the finding of conflicting results does not necessarily support that a substance that is associated with tumor development in animals is in fact a genotoxic carcinogen. Potent genotoxic carcinogens generally show patent activity in the Ames assay or in an *in vitro* chromosome aberration assays, as well as in *in vivo* studies. In addition, where DNA adducts drive the mutagenic response, adducts are found at very high levels, are persistent and show concordance with the tumor data. In the case of OTA, the data are much less amenable to straightforward interpretation. The lack of activity of OTA in the Ames assay, despite its potent effects on kidney tumor development in rats, does seem to suggest that the primary mode of action may not involve a direct genotoxic mechanism. The Ames test is highly predictive of the activity of genotoxic carcinogens (Kirkland et al., 2005). A contributory role for genotoxic activity, however, cannot be dismissed out of hand.

## DNA adduct formation

In a number of ^32^P-postlabelling studies, lesions detected have been interpreted as being OTA-derived DNA adducts in cells or cell-free extract from animals and humans (Pfohl-Leszkowicz et al., 1993; Grosse et al., 1995; El Adlouni et al., 2000), as well as *in vivo* in rodents (Pfohl-Leszkowicz et al., 1991, 1993; Faucet et al., 2004; Pfohl-Leszkowicz and Castegnaro, 2005; Manderville, 2005). In these studies, however, the identity of the DNA adducts has not been clearly established and it is not known whether in fact the alleged DNA adducts actually contain the OTA moiety. Although an OTA-DNA standard (C8-OTA-dGMP adduct) has been characterized and produced by photo-irradiation of OTA in the presence of 2-deoxyguanosine (Faucet et al., 2004) and reported to coelute with one of the spots in a ^32^P-post-labelling study in which rats were treated with OTA for 2 years (400 μg/ kg body weight thrice weekly) and pigs treated (20 μg/ kg body weight/day) for 3 weeks, this adduct could not be confirmed by subsequent ^32^P-postlabelling assays in the rat kidney *in vivo* following treatment at nephrocarcinogenic doses for 3 weeks, using stable isotope dilution LC-MS/MS methods providing for limits of detection in the range of 3.5 dG-OTA adducts/10^9^ nucleotides (Delatour et al., 2008). Mally et al. (2005a) also failed to detect this specific DNA adduct in rats treated orally with OTA at up to 2000 μg/kg body weight/day, 5 days/ week, for 2 weeks. In a number of other studies, several groups of researchers have also failed to detect any evidence of DNA adduct formation *in vitro* and *in vivo,* in some cases using similar doses as reported in the “positive” studies (Schlatter et al., 1996; Gautier et al., 2001; Gross-Steinmeyer et al., 2002; Mally et al., 2004). Recently, Mantle et al. (2010) performed an LC/MS analysis of adducts associated with the *in vitro* incubation of OTA with calf thymus DNA. On the chromatogram, the adduct cochromatographed with synthesized C-C8dG OTA. The MS analysis, based on the ions detected, was reported to demonstrate that the adduct formed with calf thymus DNA was in fact C-C8dg OTA. This adduct (synthesized) also cochromatographed with spots detected from autoradiographic analysis of the ^32^P-post labeling chromatograms of DNA extracted from the kidneys of F344 and Dark Agouti rats administered ∼6800 to 8300 μg OTA/kg body weight/day (50- to 100 fold in excess of the tumorigenic dose in the NTP study) on three consecutive days. These analyses do provide significant evidence for the presence of the C-C8dG OTA adduct in treated rats, but only at low levels (i.e., 20–70/10^9^ nucleotides). Mantle et al. (2010) also noted several potential methodological issues in studies, discussed in the previous paragraph which did not report presence of OTA DNA adducts either *in vitro* or *in vivo,* which could have led to the lack of findings of DNA adducts. Although the recent work of Mantle et al. (2010) provides compelling evidence for the presence of low levels of DNA adducts in OTA-treated rats, these researchers acknowledged that the presence of DNA adducts does not preclude the possibility of a threshold-dependent mechanism of kidney tumor formation. Moreover, the presence of DNA adducts at low levels does not necessarily lead to the conclusion that these adducts are directly involved in tumorigenesis.

Further evidence for the formation of DNA adducts *in vivo* was presented in Jennings-Gee et al. (2010). These researchers assessed the ability of OTA to induce DNA adducts in the testes of developing mice *in vivo* given that testicular and renal tissue share a common embryonic origin. In this study, three separate experiments were conducted: acute and subchronic gavage feeding, and transplacental studies. Following single gavage dosing to groups of three male BALB/c mice at 3.5, 7, 35, 70, 289, 578, or 1,056 μg/kg body weight,Jennings-Gee et al.(2010) reported, by ^32^P-post-labelling analysis, dose-dependent increases in the incidence of C-C8dg OTA adducts (from <1 to about 6 to 8 adducts/10^9^ nucleotides in each of kidney and testicular tissues. Other types of DNA adducts were also reportedly observed. In the subchronic study, groups of five male mice were administered OTA in the feed for 4 weeks to provide daily doses of 0.5, 1.4, 8, and 20 μg/kg body weight/day. Jennings-Gee et al. (2010) reported the presence of adducts in these mice as well, however, quantitative data were not presented. In the third experiment, 6- to 8-week-old pregnant Swiss mice were administered OTA by intraperitonial administration on the 17th day of gestation at a dose of 2,500 ug/ kg body weight. DNA adducts were quantified in the kidneys and testes of male newborn mice and in male mice sacrificed 1 month after birth. Three adducts were identified in newborn pups. The main spot in the kidney comigrated with C-C8 dGMP-OTA. Mean levels of this adduct in the newborns was 5.2 and 4.2 per 10^9^ nucleotides in the testis and kidney, respectively. Levels in 1-month-old mice were generally slightly higher, presumably due to suckling or release of OTA from protein binding during the period after cessation of exposure. Jennings-Gee et al. (2010) speculated that these results may support a role for OTA in the development of testicular cancer. This, however, has not been demonstrated in 2-year rodent carcinogenicity bioassays.

A causative role of DNA adduct formation in the induction of kidney tumors is difficult to ascertain given the inconsistencies in the data reported, and the discordance of low adduct levels with a high potency for kidney tumors in the male rat.

First, doses used in several of the studies with rats and mice were above those that caused carcinogenic responses in the 2-year studies (i.e., ∼170 to 2500 μg/kg body weight/day *versus* ∼50 to 150 μg/kg body weight/ day in the NTP, 1989 rat study) and exceeded estimated human exposures by upwards of 1,000,000 fold (Turesky, 2005). As a result, even if the alleged presence of DNA adducts is verified, it is not possible to extrapolate their presence to a causative role in carcinogenicity studies as these high doses may well be associated with inhibition of protein synthesis, generation of reactive oxygen species, alteration of gene transcription, cell signaling, and apoptotic processes (i.e., any DNA adducts may be artifacts rather than a direct genotoxic effect).

Second, the dose response of the reported DNA adduct formation is highly nonlinear. For example, in the studies of Pfohl-Leszkowicz et al. (1991, 1993) in which Swiss male mice were administered single doses of 600, 1,200, or 2,500 μg OTA/kg body weight, the same amounts of adducts were reported at the low and intermediate doses with a sharp increase at the highest dose where adducts, not specifically identified, were detected at a maximum of about 100 per 10^9^ nucleotides at 48 to 72 h postdosing. This was despite the fact that peak concentrations of OTA were detected in the plasma at ≤ 24 hours after treatment.

Third, the maximum levels of DNA adducts found, even at the high doses used in some of the studies, are very low and not typical of genotoxic carcinogens that produce bulky DNA adducts. Also, as discussed by Turesky (2005), the levels of DNA adducts reported in kidney samples from rats treated with OTA at 400 μg/ kg body weight/day for 2 years were low and variable in the range of <1 to 114 adducts/10^9^ bases (Castegnaro et al., 1998). Genotoxic carcinogens often produce DNA adducts at levels 100 fold (i.e., 1 to 10 adducts/10^7^ bases), or more, greater. Consistent with this fact is the report of Schlatter et al. (1996) that following single treatment of F344 rats with ^3^H-labelled OTA at a dose of 210 μg/kg body weight (i.e., the carcinogenic dose reported in the NTP, 1989 study), the covalent binding index (CBI) was calculated to be <0.25 for the kidney and <0.1 for the liver, with essentially no radioactivity detected in either organ. The CBI which is a measure of the DNA damage/dose is calculated as μmol bound chemical/mol DNA nucleotide/mmol chemical administered/kg body weight. As noted by Schlatter et al. (1996), if a direct genotoxic mechanism of action were operative, one would expect a CBI in excess of 1,000.

Finally, in the Castegnaro et al. (1998) study there was no clear correlation of the DNA adducts, which were not specifically identified as OTA adducts, with the incidence of renal tubular adenocarcinoma, and there appeared to be considerable overlap in the amounts of adducts present in male and female rats, despite the greater sensitivity of the male rat to OTA (Turesky, 2005). Also, as pointed out by Turesky (2005), the quantity of adducts reported in the kidney tissue of the 2-year study (Castegnaro et al., 1998) was lower than that reported in Swiss mice treated at 2,500 ng/kg body weight as a single dose, again despite the rat being clearly more sensitive to the nephrocarcinogenic effects of OTA.

The inconsistencies between the results of the various studies are difficult to reconcile given the differences in the methods used to isolate the DNA from tissues, differences in the solvents used in the chromatography, experimental variation in the TLC plates, pH changes, enzymatic methods for DNA hydrolysis, and so on. Beyond the conflicting nature of the data with respect to DNA adduct formation and the low levels at which any putative adducts are formed, there is also the issue of the relevance of DNA adducts to mutagenic and carcinogenic activity. The formation of DNA adducts per se does not lead to a conclusion of mutagenic activity. Only adducts that are made permanent, and which disrupt normal DNA replication, transcription, and translation processes can produce “mutagenic” effects and increase risk for cancer (Nestmann et al., 1996).

The debate surrounding the potential formation and presence of DNA adducts following exposure to OTA has been recently reviewed and its chronology summarized (Duarte et al., 2011). Overall, the role, if any, of DNA adduct formation in the carcinogenic activity of OTA has yet to be elucidated. However, the low numbers of adducts formed, in relation to the potency of OTA, would seem to be at odds with these adducts playing a major role in the induction of the carcinogenic response in the rat kidney.

## Nongenotoxic modes of action

A number of studies have been reported to provide evidence of nongenotoxic modes of action of OTA and to indicate the formation of DNA adducts as a direct genotoxic mechanism is not likely in the case of OTA (Marin-Kuan et al., 2006, 2008; Arbillaga et al., 2007; Mantle and Nagy, 2008; Mosesso et al., 2008). Examples of these are discussed below.

### Induction of oxidative stress

Several studies provide evidence for the involvement of oxidative stress in OTA-associated DNA damage in the kidneys and liver (Kamp et al., 2005; Mally et al., 2005a, 2005b). This research has shown that while no adducts were associated with OTA treatment, analysis of liver and kidney tissue in a modified comet assay showed that inclusion of formamido-pyrimidine-DNA-glycosylase (F_pg_), an enzyme known to convert oxidative DNA damage into strand breaks detectable in the comet assay, resulted in enhancement of the degree of DNA damage found. In fact, in the Kamp et al. (2005) study, which used lower doses of OTA (30, 100, and 300 μg/kg body weight/day by gavage for 4 weeks *versus* 250 to 2,000 μg/kg body weight/ day, 5 days/week for 2 weeks), no evidence of basic DNA damage was noted in the comet assay in the absence of F_pg_. Total DNA damage was only increased in the presence of the enzyme allowing for detection of oxidative DNA damage. It is unlikely to be the sole mechanism due to the fact that Mally et al. (2005a) did find that treatment of F344 rats with OTA at up to 2,000 μg/kg body weight/ day was not specifically associated with overt evidence of lipid peroxidation or an increase in the numbers of 8-oxo-7,8-dihydro-2 -deoxyguanosine (8-OH-dG) adducts, both of which are biomarkers of oxidative stress.

Palma et al. (2007) assessed OTA in the HPRT assay in Chinese hamster V79 cells and in the TK mouse lymphoma assay. In both assays, OTA was reportedly weakly mutagenic independent of metabolic activation. This is in contrast to negative results previously reported in these assays (Bendele et al., 1985; Föllman and Lucas, 2003), although treatment conditions were different (Palma et al., 2007). Although the response was weak, of note was the finding that the mutation patterns were similar to those of spontaneous mutants. This would indicate that bulky DNA adducts did not form, as under these conditions, different mutations, beyond the spontaneous types, should have been detected. The authors suggested that a slight increase in spontaneous-type mutants may be related to an increase in endogenous oxidative metabolism (Palma et al., 2007), although this has not been confirmed or refuted. It is also possible that there was only a selection for, or statistical enhancement of, spontaneous mutations rather than *de novo* mutation induction.

Arbillaga et al. (2007) reported on the results of an *in vitro* gene expression study in a human renal cell line (HK-2) which indicate that the genotoxicity associated with OTA is not due to a direct DNA-reactive mechanism. In this study, the renal cell line was exposed to OTA at concentrations of 50 μM for 6 or 24 h and gene-expression profiles subsequently analyzed. In addition, OTA was assessed for genotoxicity on the basis of the results of a comet assay conducted so as to detect oxidative DNA damage. Also, the OTA-exposed renal cell line was assessed for the presence of intracellular reactive oxygen species (ROS) by the dihydrodichlorofluorescein oxidation assay. Cytotoxicity was also assessed. The incubations resulted in slight cytotoxicity at 6 h and moderate to marked cytotoxicity at 24 h. Analysis of the gene-expression profile revealed upregulation of genes associated with mitochondrial electron transport at 6 h and, additionally, genes associated with oxidative stress conditions (e.g., inflammation response, calcium regulation and complement and coagulation cascades) after 24 h. At both time points, intracellular ROS and oxidative DNA damage (comet assay) were increased and were greater as the degree of cytotoxicity increased. Noteworthy was the lack of any finding of upregulation of genes associated with cellular processes involved with DNA damage (e.g., apoptosis, cell cycle control), even at the 24-h exposure point. These data are all consistent with a mechanism of action not involving direct genotoxicity. Furthermore, these data also support a role of oxidative stress/ROS generation in the “genotoxic” response of OTA. The reported presence of DNA adducts in some studies may represent nonspecific oxidative adducts (EFSA, 2006) which are formed indirectly due to oxidative stress or oxygen radical formation at the high-dose levels employed in many of the studies (Mosesso et al., 2008).

Marin-Kuan et al. (2006) conducted a toxicogenomic study in which groups of male F344 rats were dosed with OTA (∼100 μg/rat) for 7 days to 12 months. Gene-expression profiles were assessed at various intervals. Tissue-specific responses were observed in the kidney versus the liver. In the kidney, the expression of several genes known as markers of kidney injury and cell regeneration was significantly altered, either up or downregulated, by OTA. Of significance was the finding that the expression of genes known to be involved in DNA synthesis and repair, or of genes induced as a result of DNA damage, was only marginally altered by OTA treatment. Also, expression of genes thought to be linked with programmed cell death (apoptosis) appeared to be little, if at all, affected by OTA treatment. Alterations of gene expression indicating effects on calcium homeostasis were noted. This finding was consistent with the results in the human renal cell line reported by Arbillaga et al. (2007). Interestingly, the genes assessed that were relevant to oxidative stress and xenobiotic metabolism were generally downregulated by OTA treatment. The gene expression profiles of elements regulated by hepatocyte nuclear factor 4α (HNF4α) and nuclear factor-erythroid 2-related factor 2 (Nrf2) were reported to occur in the kidney, but not in the liver. Marin-Kuan et al. (2006) noted that a reduction in HNF4α may be associated with nephro-carcinogenicity. Also, these researchers reported that Nrf2-regulated genes are involved in detoxification and antioxidant defense processes. Reduced activity/ output of these genes could well be associated with impairments to the defense mechanisms in kidney cells, resulting in chronic elevation of oxidative stress. Such oxidative stress could, theoretically, be involved in the carcinogenic response of the kidney to OTA (Marin-Kuan et al., 2006; Cavin et al., 2007). Also, in their study, there were other observed changes in gene expression which support an epigenetic rather than a genotoxic mechanism of action (Marin-Kuan et al., 2006, 2008). Cavin et al. (2007) also report that reductions in Nrf2-regulated genes noted in the Marin-Kuan et al. (2006) study were associated with analogous expression of proteins under Nrf2 genetic/regulatory control; therefore, the results reported by Marin-Kuan et al. (2006) are likely of biological significance. In addition, Cavin et al. (2007) reported an increased formation of oxidative DNA damage, as measured by the number of abasic sites, both *in vitro* (rat NRK kidney cells) and *in vivo* (kidneys from OTA dosed rats), further implicating a role of oxidative stress/reduced antioxidant defense mechanisms in the effects of OTA on the kidney.

### Additional epigenetic mechanisms

Several review articles (Schilter et al., 2005; JECFA, 2008; Mally and Dekant, 2009) document a complex array of epigenetic mechanisms that may play critical roles in the development of kidney tumors and DNA adducts in rodents treated with OTA.

Potential mechanisms by which OTA may induce indirect genotoxic effects include the following: cell proliferation; alterations to cellular apoptosis (Zhang et al., 2009); changes in gene expression (Adler et al., 2009); disruption of cell cycle progression (Adler et al., 2009); perturbation of mitosis (Rached et al., 2006; Czakai et al., 2011), alterations to cell-cycle signal transduction; protein-synthesis inhibition; mitochondrial dysfunction; and activity of mitogen-activated protein kinases (MAPKs; Mally and Dekant, 2009).

The complex nature of the epigenetic mechanism(s) that appears to be involved in OTA-associated carcinogenicity, and likely in adduct formation as well, precludes the teasing out of any one as a singular definitive cause. In summary, the results of classical Organisation for Economic Co-operation and Development-compliant type mutagenesis studies on OTA have been mixed. In standard Ames assays OTA is nonmutagenic, whereas *in vitro* studies with mammalian cell lines have shown negative results or only weak evidence of mutagenic effects. *In vitro,* DNA strand breaks have been recorded with OTA, however, frank clastogenic effects appear only associated with cytotoxicity. The lack of activity of OTA in the Ames assay is unusual for a genotoxic carcinogen or of DNA-adduct directed genotoxicity. Overall, the majority of evidence favors a non-DNA reactive mechanism(s) not involving classical genotoxic effects.

### Dose response assessment

Doses of OTA, or exposure limits, determined to be without appreciable risk of adverse effects, have been derived by regulatory agencies and authoritative bodies. These exposure limits vary by about five fold depending on whether the basis of the exposure limit is the lowest observed adverse-effect level (LOAEL) determined from the 90-day pig study (Krogh et al., 1974) or the tumor data determined from the NTP rat study (NTP, 1989) and assuming a nonthreshold mechanism. Examples of recently derived exposure limits representative of these two different methods are by the EFSA (2006) and Health Canada (Kuiper-Goodman et al., 2010).

EFSA (2006) concluded that the mechanism for carcinogenic response of OTA was most likely threshold based, the significance being that low doses below the threshold dose would not be associated with a cancer risk. A TWI of 120 ng/kg body was derived for OTA from the LOAEL of 8 μg/kg/day for renal toxicity in pigs by applying an uncertainty factor of 450 to account for toxicodynamic interspecies differences between the pig and human (2.5 fold), kinetic differences based on the half-life of OTA (six fold), intraspecies variability (10 fold), and the use of a LOAEL instead of a NOAEL (three fold). On a daily basis, the TWI would correspond to approximately 17 ng/kg body weight/day.

Using an approach comparable to EFSA (2006), JECFA (2008) derived a provisional tolerable weekly intake (PTWI) for OTA of 100 ng/kg body weight, or approximately 14 ng/kg body weight/day, based on the pig study.

Kuiper-Goodman et al. (2010) concluded that the genotoxic status of OTA is equivocal and recommended the default approach that OTA be regulated as a nonthreshold carcinogen. A negligible cancer-risk intake (NCRI), of 4 ng/kg body weight/day, defined as the exposure associated with a risk level of 1:100,000 was derived for OTA. As the first step in calculating the NCRI, Kuiper-Goodman et al. (2010) determined the tumorigenic dose associated with a 5% increase in tumor incidence above background (TD_05_) using the multistage model. The tumor incidence data for the male rat from the NTP gavage study were used in the model. The resultant TD_05_ was adjusted downward from 27.4 to 19.6 μg OTA/kg body weight/day as exposure to OTA in the NTP study was by gavage on only 5 out of 7 days (27.4 × 5/7= 19.6). The NCRI was then derived by dividing the TD_05_ by 5,000, which was considered representative of linear extrapolation to zero exposure.

It could be argued that basing the TD_05_ on the gavage NTP study results for the male rat is overly conservative for two primary reasons: (i) there is a large disparity between renal tumor incidence data based on species and gender, which indicates that male rats may be unusually sensitive to OTA; (ii) the gavage administration of OTA has been demonstrated to be associated with a greater increased renal tumor incidence than that observed following dietary administration, which is the exposure route for humans. This has been demonstrated with the same doses of OTA given by gavage (as in NTP study) versus in the diet (mimicking human exposure; Miljkovic et al., 2003; Mantle et al., 2005).

Although OTA had previously been discounted as a classical α-2u-globulin-associated renal carcinogen, recent research efforts have revisited this issue and demonstrated that there may be a contributory role of α-2u-globulin in the male tumor response to OTA (Mantle and Nagy, 2008). As α-2u-globulin is not present in humans, deferring to a TD_05_ based on the female data, which would be the more representative model for human relevance, would result in a higher NCRI. Based on the renal tubule cell tumor incidence data for the female rat, the adjusted TD_05_ (0.05) could be expected to fall slightly above 50 μg/ kg body weight/day given the observed tumor incidence data. Thus, the adjusted NCRI (if TD_05_ is 50 μg/kg/day), would be greater than 10 ng/kg body weight/day (as opposed to 4 ng/kg body weight/day based on the male rat).

As a side evaluation, Kuiper-Goodman et al. (2010) recalculated the tolerable daily intake (TDI) from the 90-day pig study (Krogh et al., 1974) using a different methodology than EFSA (2006). Instead of using the LOAEL of 8 μg/kg body weight/day, which was associated with effects on renal enzymes and renal function, as starting dose and applying an uncertainty factor of three [as per EFSA (2006) methodology], Kuiper-Goodman et al. (2010) derived a benchmark dose (BD_10_) of 1.56 μg/kg body weight/day from the pig study (Krogh et al., 1974) for use as the starting dose. Both used an uncertainty factor of 10 for intraspecies differences. Kuiper-Goodman et al. (2010) applied an additional uncertainty factor of two to extrapolate the 90-day subchronic study to a chronic study (this is a default that is consistent with other risk-assessment methodologies). However, in other longer term pig studies, such as the follow-up study by Krogh et al. (1979), the exposure of pigs to approximately 40 μg/kg body weight/day for up to 2 years led to progressive nephropathy but no renal failure (EFSA, 2006). Hence, the changes in renal function occurring at the lower dose of 8 μg/kg body weight/day in the 90-day study would not have been expected to progress to renal failure should exposure have been continued. Thus, the additional uncertainty factor of two should not be necessary. The uncertainty factors used for total interspecies differences were 15 and 25 for EFSA (2006) and Health Canada, respectively. Both EFSA (2006) and Kuiper-Goodman et al. (2010) adjusted the toxicokinetics component of the uncertainty factor to account for differences in half-life as discussed below.

EFSA (2006) applied a total uncertainty factor of 450 to the LOAEL (starting dose) of 8 μg/kg body weight/ day to obtain a TDI of 17 ng/kg body weight/day, whereas Kuiper-Goodman et al. (2010) applied a total uncertainty factor of 500 to a starting dose of 1.56 μg/ kg body weight/day to obtain a TDI of 3 ng/kg body weight/day. However, use of differences in half-lives to increase the interspecies uncertainty factor to 25 (or to 15, as was done by EFSA), is not fully justified. Given that OTA is highly protein bound in the plasma, there is no clear evidence that a longer half-life increases sensitivity, but that it could actually be an indication of the contrary. The best means of predicting differences between species remains to be elucidated. One area to investigate is whether or not the strength of binding to plasma protein, while increasing circulation time, may show an inverse relationship to toxicity, given the lack of residence time in target organs.

Use of only the half-life to adjust the interspecies uncertainty factor also is questionable since the human half-life value is based on a single volunteer and an adjustment for percent protein binding of OTA was not likewise considered. The amount of unbound OTA in human plasma was 0.02% compared to 0.1% for the pig. Simplistically, if both the half-life and plasma binding differences were considered in calculating the uncertainty factor for the intraspecies difference between the pig and the human, the uncertainty factor would be only five, as per the following relationship:

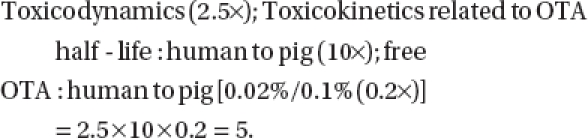
 Assuming the other uncertainty factors discussed above (i.e., intraspecies, LOAEL or no observed adverse-effect level or Benchmark dose, subchronic to chronic) remained the same, use of five as the new interspecies uncertainty factor would result in TDI values of 53 ng/ kg body weight/day and 15.6 ng/kg body weight/day, in place of the TDI values reported by EFSA (2006) and Kuiper-Goodman et al. (2010), respectively. Also, although the conservative default in risk assessment is to assume that the human is the most sensitive species for any toxicant, there are no data for humans to suggest a greater sensitivity to OTA than for pigs.

### Exposure assessment

Exposures to OTA were estimated by Kuiper-Goodman et al. (2010) using various mathematical models. In an effort to account for the highly variable occurrence data and to provide more realistic estimates of exposure, multiple methods of analysis were employed and compared. These included a partial probabilistic and a full probabilistic (Monte Carlo) approach, the latter which was further adjusted to examine usual exposure estimates. Monte-Carlo is a stochastic mathematical modeling approach that allows aspects of variability and uncertainty to be considered in the estimation of exposure. In this case, two main approaches were used, first a “partial” probabilistic exposure assessment was used, which multiplied distributions of “all person” food-consumption data by the mean OTA-occurrence data. The second approach was the “full” probabilistic exposure, which combined distributions of food-consumption data with distributions of OTA in foods. The results were presented on the basis of age–sex strata for the mean and various percentiles of exposure (Kuiper-Goodman et al., 2010). Confidence interval data (5th and 95th) also were presented. No “all ages”, or “total population” data were presented.

Various approaches to the input parameters and treatment of the exposure models, specific to the present case of estimating exposure to OTA were used by Kuiper-Goodman et al. (2010) and are outlined as follows:

1. Occurrence data: In this assessment, Canadian occurrence data for raw food commodities and various finished foods gathered over the past decade were used. OTA-occurrence data used in the exposure assessment were available for 37 different food commodities or categories that are known to sometimes contain OTA. Some of these commodities were rice, breakfast cereals (including corn, multigrain, oat, rice, and wheat-based cereals), infant cereal, pasta, raisins, wheat (hard, soft, and durum), oats, barley, and peas. Beverages included beer, wine, grape juice, and coffee (ground-regular and decaffeinated, and instant-regular and decaffeinated).
2. Processing factors: To account for redistribution of OTA during the milling process, processing factors were used to convert the concentration of OTA in the raw grain to the foods as processed.
3. Handling of censored data: Two methods were used to deal with occurrence values that fell below the limits of detection/quantification, including a distribution developed by the authors, and by using ½ the LOD or ½ the LOQ as appropriate.
4. Maximum Levels (ML): In Europe maximum levels (ML) have been set for OTA in many food commodities, and these ML's were also used in the exposure models. When the MLs were used in the models, OTA occurrence values above these levels were assigned values equal to these levels (“ML-modified distribution of occurrence.”)
5. Food consumption data: The age–sex strata refer to representative population groupings based on the reported ages and gender of the dietary recall survey respondents. The U.S. Continuing Survey of Food Intakes by Individuals (CSFII) food intake survey 1994–1996 and 1998 (USDA, 2000), is the basis of the exposure estimates, and includes food consumption data from two nonconsecutive survey days for >20,000 persons, of all ages. Validation procedures were run to ensure these data were appropriate for Canada.

The full probabilistic usual (P*P) mean and 90th percentile exposures to OTA that were reported by Kuiper-Goodman et al. (2010) are included in Table 3. The data were determined on a kg body-weight basis and were reported for all persons on an age–sex stratum.

**Table 3:** Comparison of estimated dietary OTA exposures to LOAEL and TD_05_ for all persons (ng/kg bw/d).

Age	Mean	MoE for LOAEL of 8,000 ng/kg/day	MoE for TD_05_of 19,600 ng/kg/day	p90	MoE for LOAEL of 8,000 ng/kg/day	MoE for TD_05_ of 19,600 ng/kg/day
0-2 months	2.22	3,604	8,829	6.07	1,318	3,229
3-5 months	1.93	4,145	10,155	5.18	1,544	3,784
6-8 months	2.45	3,265	8,000	5.88	1,361	3,333
9-11 months	3.45	2,319	5,681	7.43	1,077	2,638
1 year	4.38	1,826	4,475	8.66	924	2,263
2 years	4.36	1,835	4,495	7.88	1,015	2,487
3 years	4.22	1,896	4,645	7.81	1,024	2,510
4 years	3.96	2,020	4,949	7.16	1,117	2,737
5-6 years	3.66	2,186	5,355	6.77	1,182	2,895
7-11 years	2.6	3,077	7,538	4.72	1,695	4,153
12-18 years	1.76	4,545	11,136	3.25	2,462	6,031
19-30 years M	1.76	4,545	11,136	3.4	2,353	5,765
31-50 years M	1.62	4,938	12,099	3.06	2,614	6,405
51-70 years M	1.43	5,594	13,706	2.73	2,930	7,179
71+years M	1.33	6,015	14,737	2.58	3,101	7,597
12-18 years F	1.41	5,674	13,901	2.53	3,162	7,747
19-30 years F	1.33	6,015	14,737	2.54	3,150	7,717
31-50 years F	1.33	6,015	14,737	2.54	3,150	7,717
51-70 years F	1.23	6,504	15,935	2.35	3,404	8,340
71+years F	1.15	6,957	17,043	2.23	3,587	8,789

LOAEL = lowest observed adverse effect level; TD_05_ = tumorigenicdose associated with a 5% increase in tumor incidence above background.

A full probabilistic exposure involves a scenario in which both the intake of foods and levels of OTA are modeled as distributions. These distributions are then employed in a Monte Carlo analysis in which an individuals' daily exposure to OTA is estimated many times, with each estimate run known as an iteration. In this case Kuiper-Goodman et al. (2010) ran 1,000 iterations.

Along with the full probabilistic model of exposure, an adjustment was made to develop a “usual” model of exposure. This model was developed by the authors and based on adaption of previous “usual” exposure models developed for nutrients. The usual exposure model extrapolates exposure from a short-term survey (e.g., 1 or 2 days) to a longer, more usual expectation of exposure. For the usual exposure model, both the “within” and “between” components of variance need to be considered. In this case, a “one-day” model was chosen, where the food-intake data were selected from either of the two survey days in the CSFII data to increase the realism of the model.

Although the results presented in Table 3 are for “all persons,” the data are essentially the same as for eaters only (consumers of any potentially OTA-containing commodity), as for all age groups from 6 to 8 months up to 71+ years, 97% to 100% of all persons included in the survey consumed at least one potentially OTA-containing commodity (are “eaters”; from the age range of 9 to 11 months to 71+ years, between 99.4% to 100% are eaters). For the earliest age groups of 0 to 2 months and 3 to 5 months, the percentage of surveyed individuals that are eaters, are 43% and 74.5%, respectively. For the 1-year age group, out of 1,040 respondents, 1,035 (99.5%) were eaters with the majority, 1,021 respondents (98.6%) having consumed foods that could potentially contain OTA on both survey days.

For the full probabilistic-exposure model, during a single iteration, the workings of the model are as follows. Based on the underlying food intake distributions for each individual, the software selects one input per food group, similarly based on the underlying distributions for occurrence data, the software selects one value per food group per individual. These values were combined and this process was repeated 1,000 times, producing an exposure distribution of 1,000 values per individual. These exposures per food group were summed across all food commodities consumed by that individual to produce their total estimated OTA exposure on that survey day. This process was repeated for the entire population, which produced an overall exposure distribution. This distribution was broken down according to the age and sex of the individuals, and described by calculating certain distribution parameters (mean, 50th, 75th, 90th, and other percentiles of exposure). The 2-day average of these overall results gave a rough estimate of chronic exposure.

In completing the exposure assessment, the following assumptions were necessary.

- Many of the concentration data are based on raw food commodities and it was necessary to apply a processing factor, to account for known redistribution of OTA during the milling process, to estimate concentrations in finished products. Assuming a processing factor rather than basing the concentrations on only finished foods introduces additional uncertainty into the process. The processing factors used to correct the data were very conservative as the actual analytical data for pasta contamination were lower than that derived using the data for durum wheat as a surrogate and the estimated processing factors of 0.82 or 0.64 for the upper-bound and lower-bound estimate, respectively.
- Food samples for which OTA was not detected were not assumed to contain zero OTA. Such samples were usually assigned a concentration value based on the lognormal distribution of known concentrations or based on half of the limit of detection/quantification. The first method was preferred as the latter method tends to overestimate the mean when the number of positive samples is small.
- The correction of the 2-day survey data to represent “usual” intake or intake over significant time periods (i.e., 3 months to 1 year) is originally based on statistical methods for determining nutrient intake. Although this procedure may be statistically correct, in practice, the amount of correction is generally in the range of two fold whereas the difference in food intake using longer time data (e.g., up to 14-day data) suggests up to a 10-fold difference in the intake of foods that are not consumed on a daily basis.

As stated by Kuiper-Goodman et al. (2010), mycotoxin concentrations within a particular food commodity can vary by several orders of magnitude. Therefore, the exposure estimates for the upper percentiles (95th, 97.5th, and may be even the 90th) are based on iterations that combined the upper range of food consumption data and the highest contaminant estimates of the input ranges. These data may be useful for the assessment of acute health risks, but would not be reflective of chronic intakes as it is unrealistic to expect that the highest consumers would always eat the most contaminated foods. The exposure estimates at these percentiles should not be used for predicting health risks associated with chronic OTA exposures. Likewise, OTA food standards if derived by taking into consideration the results of Margin of Exposure calculations using exposures at these upper-bound percentiles, may be unnecessarily conservative.

Two additional studies reporting on the concentrations of OTA in foods sold in Canada were recently published (Bansal et al., 2011; Tam et al., 2011). In the first study, 200 samples of rice including both domestically grown and imported samples were analyzed for the presence of OTA (Bansal et al., 2011). The average concentration of OTA in the rice samples over the 2 years studied were 0.05 and 0.005 ng/g, respectively. The limit of detection (LOD) was 0.05 ng/g; therefore, the estimated concentration in the second year of study was less than the LOD and the only sample above the LOD was an imported sample of rice with an OTA concentration of 0.49 ng OTA/g. All samples were below the proposed ML of 5 ng/g. In comparison, the mean occurrence value of OTA in rice reported in the Kuiper-Goodman et al., (2010) exposure assessment was 0.80 ng/g with no ML and 0.68 ng/g when positives above the European ML were set to the ML.

The Tam et al. (2011) study reported results from the Canadian Total Diet Study from foods collected in Quebec City in 2008 and from Calgary in 2009. This study involved the analysis of numerous different food products and composites for the presence of OTA. Although 73% of samples had levels of OTA above the LOD (which ranged from 0.001 ng/g 0.008 ng/g, depending on the food stuff), the highest measured OTA value from the Quebec city survey was a sample of raisins containing 2.3 ng OTA/g and the highest measured value from the Calgary survey was a sample of wheat flour containing 1.7 ng/g, both of which are below the proposed ML.

### Risk characterization

Given the disagreement between agencies as to what would constitute a dose of OTA that would be acceptable, for the purpose of this review, consumption estimates were compared directly to the pig LOAEL and the rat TD_05_. The average consumption estimates are considered representative of chronic dietary intakes whereas the 90th-percentile (p90) estimates may be more comparable to acute dietary intakes such as could occur with problematic climate or storage conditions. The margin of exposure (MoE) comparison by age group is provided in Table 3.

Dietary OTA exposures at the mean and 90th percentile are three to four orders of magnitude below the LOAEL determined from the pig dietary study and the TD_05_ determined from the rat gavage cancer study. Furthermore, these estimates of exposure are considered to be conservative or worst case as much of the concentration data was based on raw food commodities. This necessitated the assumption of a processing factor which introduces additional uncertainty.

The data provided by Kuiper-Goodman et al. (2010) also allow for calculation of the resultant theoretical reduction in risk with the implementation of standards. Kuiper-Goodman et al. (2010) estimated what the intakes would have been if maximum levels (ML) from the European Commission (EC; Commission of the European Communities, 2006) had been in effect and consistently met in all foods during the data collection period. This was accomplished by using the EC ML as the maximum concentration for all of those samples that exceeded that level. The TD_05_ estimate was divided by the estimates of exposure to determine a MoE. A ratio equal to 5,000 was considered to correspond to a risk of developing renal cancer of 1:100,000. If the MoE was less than 5,000, it was concluded that there was a high priority for risk reduction. The MoE calculation was used for individual life stages rather than for lifetime exposures with no adjustment for consideration of duration of exposure. An MoE of 2446 was reported at the 90th percentile all consumers intake for respondents 1 year of age. When the EC ML was applied the MoE increased to 3289. Thus, if the MoE of 5,000 was associated with a risk level of 1-in-100,000, the MoE of 2,446 would theoretically be associated with a risk level of 1-in-48,920. This would be the expected risk if the individual remained at the same weight as a 1-year-old for a lifetime (usually considered 70 years), and continued to eat the same diet as the 1-year-old for a lifetime. If EU maximum limits for OTA were adopted, the MoE of 3,289 would theoretically be associated with a risk level of 1-in-65,780, again if the individual remained the same weight, and ate the same diet as a 1-year-old for a lifetime.

For both the MoE values of 2,446 and 3,289, the risk level is much less than the estimated lifetime cancer risk associated with ingestion of drinking water containing arsenic, a known human carcinogen, at the Health Canada-adopted Maximum Acceptable Concentration (MAC) of 10 μg/L (Health Canada, 2006). The lifetime cancer risk levels for arsenic at the MAC range from 1 in 33,333 to 1 in 2,564, depending on the tumor type. The risk level for a 1 -year-old is not reported.

Although the arsenic cancer-risk levels at the MAC are above what Health Canada considers to be “essentially negligible” (i.e., 1 in 100,000 to 1 in 1,000,000), Health Canada has justified adoption of the arsenic MAC as a risk-management decision as it exceeds the health-based guideline value. In accepting this MAC, Health Canada (2006) decided that it represents “the lowest level of arsenic in drinking water that can be technically achieved at reasonable cost, especially for smaller public systems and private wells.”

Similar consideration is not given to OTA which also is a natural contaminant. Given the vast quantities of foods that are produced and imported into Canada, it is probably impossible for the affected agriculture and food industries to be able to guarantee that all shipments to which proposed OTA standards would apply will meet the proposed standards. The concentration data that Kuiper-Goodman et al. (2010) used in their exposure assessment, support that the majority of grains produced in Canada under current agricultural practices, and foods marketed, have very low OTA levels. The commodity with the greatest number of samples exceeding the proposed standard was infant cereal (26 >ML out of 296), however, this was more attributable to the standard for finished infant foods being 10 fold lower that the raw grain standards (0.5 ng/g versus 5 ng/g). The infant food standard would appear to be unnecessarily stringent given the very short time that the population consumes such foods. Most children are consuming adult foods by age of 1 year. Also, a processing factor of 0.1 (reducing or redistribution effect) would be needed to obtain finished infant cereals from the proposed maximum limit for raw grains. In comparison, the lower bound processing factor selected by Kuiper-Goodman et al. (2010) for use in the exposure assessment was 0.64 (corresponds to a 36% reduction in levels of OTA in raw grain versus finished foods). This gap suggests that consistent compliance with the proposed ML of 0.5 ppb is not reasonably achievable. Up to 8.78% of finished commodities and up to 14.81% of raw commodities, based on survey data between 1993 and 2006, exceeded the ML (Kuiper-Goodman et al., 2010). It remains to be seen if future enforcement actions of adopting standards would include destroying these lots. If these percentages of samples exceeding the ML are representative of future out of compliance product/ commodities that reach the market and/or are discarded early in the process, the implications could be quite serious including forcing companies out of business or substantially reducing crop yields.

### Derivation of lifetime MoE for OTA

The MoE is the difference between the dietary exposure and the dose in rats associated with a 5% incidence of tumors above background (incidence in control or non-treated rats). Thus, the larger the MoE, the greater the confidence that no adverse effects will occur.

Kuiper-Goodman et al. (2010) indicated that when the difference between the mean or 90th percentile intakes and TD_05_ equals 5,000, the risk of developing renal cancer is taken as 1:100,000. The NCRI is similar in concept to other exposure limits such as the no significant risk level (NSRL), of the California Environmental Protection Agency, or a 1 in 100,000 risk-specific dose (RsD) level based on a calculated slope factor, as per the U.S. Environmental Protection Agency (U.S. EPA) Integrated Risk Information System (IRIS) methodology. However, these other values are representative of a lifetime risk. Thus, a MoE < 5,000 for a single age group would not truly reflect the incidence of 1:100,000 lifetime cancer risk above background.

There is an inverse relationship when discussing the MoE and lifetime cancer risk. Hence, the greater the MoE, the lower the cancer risk.

The approach of applying of a factor of 5,000 to the TD_05_ to estimate the NCRI is discussed in greater detail in Kuiper-Goodman (2004). Two additional important points made in Kuiper-Goodman (2004), one of which was applied to this assessment and one that was not, are highlighted below:

This “approach can also be used for those threshold carcinogens for which there is some uncertainty regarding the mode of action (threshold *versus* non-threshold, e.g. Ochratoxin A)”

The “factor of 5000 can be decreased when additional biological information on the mode of action or quantitative aspects relating to human relevance indicate less concern”.

Kuiper-Goodman et al. (2010) did not present the MoE for total population lifetime risks, but rather used a lifetime exposure limit for short-term exposures during specific periods of a lifetime. Using the Kuiper-Goodman et al. (2010) individual age group MoE values, we have extrapolated lifetime cancer risks, as summarized in Table 4, for OTA in rice and in hot oatmeal, and total population 90th-percentile intakes. The lifetime MoE for each category is calculated by multiplying the number of years in each age group by the age-specific MoE, summing the resulting totals for all age groups and dividing the grand total by 70 years.

**Table 4:** Calculation of lifetime MoE using Kuiper-Goodman et al. (2010) data for specific commodities and all commodities.

Age (years)	No. of years	Rice MoE (no ML)	No. of years × MoE	Hot oatmeal (no ML)	No. years × MoE	All commodities (per age strata) p90(no ML)	No. of years × MoE
0 to 6^a^	6	3,972	23,832	2,188	13,128	2,446	14,676
7 to 11^b^	5	6,588	32,940	3,918	19,590	4,360	21,800
12 to 18^c^	7	8,767	61,369	5,633	39,431	6,306	44,142
19 to 30^d^	12	9,611	115,332	5,821	69,852	6,399	76,788
31 to 50^e^	20	12,384	247,680	8,563	171,260	8,230	164,600
51 to 70^f^	20	12,384	247,680	8,563	171,260	8,230	164,600
Lifetime MoE^g^ (sum of group MoE/70 years)	70		10,412		6,922		6,952

a MoE values are those reported in Kuiper-Goodman et al. (2010) for the 1 year age group with no ML.

b MoE values are those reported in Kuiper-Goodman et al. (2010) for the 7 to 11 year age group with no ML.

c MoE values are those reported in Kuiper-Goodman et al. (2010) for the 12 to 18 year age group with no ML.

d MoE values are those reported in Kuiper-Goodman et al. (2010) for the 19 to 30 year age group with no ML.

e MoE values are those reported in Kuiper-Goodman et al. (2010) for the 31 to 50 year age group with no ML.

f MoE values for the 51 to 70 year age group are the same as for the 31 to 50 year age group which is conservative since OTA exposures for the older adult strata are lower than the middle age strata reported in Kuiper-Goodman et al. (2010) with no ML.

g The lifetime MoE is calculated by multiplying the number of years in each age group by the age-specific MoE, summing the resulting totals for all age groups and dividing the grand total by 70 years; the lifetime MoE was calculated by Cantox; other data were from the Kuiper-Goodman et al. (2010) publication.

One of the first foods introduced to children is rice. Thus, using the Health Canada-generated MoE values for regular specific commodity eaters (RCE) of rice as an example, a lifetime MoE adjusted for duration of exposure over a 70-year lifetime has been estimated. A lifetime MoE also has been estimated for hot oatmeal regular eaters, which had the lowest reported MoEs, and for 90th percentile all-person exposures. It should be noted that although intakes can be determined for all age groups, Kuiper-Goodman et al. (2010) did not report results for ages 2 through 6. Thus, for the purpose of determining the lifetime MoE analysis, MoEs for this age group have been assumed to be the same as the values for 1-year-olds. This is overly conservative as body weights increase significantly between ages one and seven. For the 51- to 70-year age group, also not reported, MoE values for the 31- to 50-age group were applied as OTA exposures presented by Kuiper-Goodman et al. (2010) were lower for the older adult strata (51 to 70 years), than for the middle-aged strata (31 to 50 years). The results of this analysis, as estimated lifetime MoEs, are provided in Table 4.

In all cases, the lifetime MoE (difference between human exposure and the TD_05_) is greater than 5,000 (i.e., cancer risk level is less than 1 in 100,000), even without adjusting the higher concentration data reported downward to the EC ML.

Although the results for all commodities are for “all persons,” the data are essentially the same as eaters only (consumers of any potentially OTA-containing commodity), as the data in the publication indicate that for all age groups from 6 to 8 months up to 71+ years, 97% to 100% of all persons included in the survey consumed at least one potentially OTA-containing commodity (are “eaters”; from the age range of 9 to 11 months to 71+ years, between 99.4% to 100% are eaters). For the earliest age groups of 0 to 2 months and 3 to 5 months, the percentage of surveyed individuals that are eaters, are 43% and 74.5%, respectively. For the 1-year age group, out of 1,040 respondents, 1,035 (99.5%) were eaters with the majority, 1,021 respondents (98.6%) having consumed foods that could potentially contain OTA on both survey days.

It should be noted that no data, such as plasma levels in infants, plasma half-lives for OTA or urinary clearance rates are available to support greater sensitivity of children to the effects of OTA. In fact, the data from the recent feeding study conducted with male Dark Agouti rats (20/group; Mantle, 2009) demonstrated that 3 months continuous exposure to 5 ppm OTA (doses ranging from 640 μg/kg/day at commencement and declining to 450 μg/kg at 3 months inversely, according to growth) was not associated with an increased incidence of renal tumors or other obvious pathological changes at the end of the 2-year observation study period. In the groups of male rats administered 5 ppm OTA for 6 months, and 9 months, 1 out of 20, and 4 out 20 rats, respectively, developed renal tumors before the end of the study. The incidence of renal tumors was not increased in Dark Agouti rats administered 400 ppb OTA (∼50 μg/kg body weight/day decreasing to 20 to 30 μg/kg body weight/day for adults) from 8 weeks of age throughout natural life.

As the MoEs in Table 4 are based on the exposure data prior to correcting for EU ML standards, it can be inferred that the foods available in Canada, even without adoption of proposed maximum limits, have been of acceptable quality in terms of dietary exposure to OTA.

However, as there is uncertainty regarding the sensitivity of children to chemicals and exposures are greater on a body-weight basis, risk-assessment practices have been developed to determine how early life exposures might impact lifetime cancer risks. The U.S. EPA Office of Research and Development has been at the forefront of risk assessment and cancer-evaluation methodology development for many years and it is an important resource for obtaining cancer potency data for specific chemicals determined to be human carcinogens. Potential human exposures may be very different than the route and dosing schedule employed in animal studies from which the slope factors have been derived. Thus, to account for intermittent or varying levels of exposure experienced by humans, the U.S. EPA has recommended calculating a lifetime average daily dose (U.S. EPA, 2005a). This recommendation is based on the assumption that the risk associated with a high dose of a carcinogen received over a short period of time is equivalent to a low dose spread over a lifetime. This also implies that less than lifetime exposure is associated with a linearly proportional reduction of the lifetime risk. Thus, assessment of less than lifetime human exposures has also employed the use of a lifetime average daily dose, which is adjusted to account for the number of years of exposure divided by 70 years (considered lifetime). However, additional research has indicated that this reduction in risk may not be directly proportional due to susceptible populations and life stages (Murdoch et al., 1992; Halmes et al., 2000). Consequently, a modifying factor may need to be applied to the cancer-risk estimate depending on the circumstances to ensure risks are not underestimated.

In the supplement to the guidance document (U.S. EPA, 2005b), the U.S. EPA calculated age-dependent adjustment factors (ADAFs) to account for the possibility that children are more susceptible to carcinogens. These factors, the values of which are indicated below, are applicable to carcinogens with a genotoxic mode of action as opposed to apparent threshold carcinogens such as OTA.

- Children from birth to <2 years of age: ADAF = 10
- Children from 2 to < 16 years of age: ADAF = 3
- 16 years of age: ADAF = 1 (i.e., no adjustment).

It is noted that the EPA approach is relevant to chemicals with a mutagenic mode of action (MoA) for carcinogen-esis which is probably not the case for OTA.

Nevertheless, we repeated the calculation of lifetime MoEs from Table 4 to take into consideration the impact of early childhood sensitivity on lifetime risk. As the age breakdown in Table 4 does not correspond to the EPA age divisions for the application of ADAF, the calculations were adjusted accordingly (as described in the footnotes to the following table). The results are summarized in Table 5.

**Table 5:** Calculation of lifetime MoE using Kuiper-Goodman et al. (2010) data for specific commodities and all commodities with age-dependent adjustment factors (ADAF) applied.

Age (years)	No. of years	Rice MoE (no ML)	No. of years x MoE	Hot oatmeal (no ML)	No. of years x MoE	All commodities p90(no ML)	No. of years x MoE
0 to 2^a^	2	397	794	219	438	245	490
3 to 6^b^	4	1,324	5,296	729	2,917	815	3,261
7 to 11^c^	5	2,196	10,980	1,306	6,530	1,453	7,267
12 to 16^d^	5	2,922	14,612	1,878	9,388	2,102	10,510
17 to 18^e^	2	8,767	17,534	5,633	11,266	6,306	12,612
19 to 30^f^	12	9,611	115,332	5,821	69,852	6,399	76,788
31 to 50^g^	20	12,384	247,680	8,563	171,260	8,230	164,600
51 to 70^h^	20	12,384	247,680	8,563	171,260	8,230	164,600
Lifetime MoE^l^ (sum of group MoE/70 years)	70		9,427		6,327		6,288

a MoE values are those reported in Kuiper-Goodman et al. (2010) for the 1 year age group with no ML but have been divided by a ADAF of 10.

b MoE values are those reported in Kuiper-Goodman et al. (2010) for the 1 year age group with no ML but have been divided by a ADAF of 3.

c MoE values are those reported in Kuiper-Goodman et al. (2010) for the 7 to 11 year age group with no ML but have been divided by a ADAF of 3.

d MoE values are those reported in Kuiper-Goodman et al. (2010) for the 12 to 18 year age group with no ML but have been divided by a ADAF of 3.

e MoE values for the 51 to 70 year age group are the same as for the 31 to 50 year age group which is conservative since OTA exposures for the older adult strata are lower than the middle age strata reported in Kuiper-Goodman et al. (2010) with no ML and no ADAF applied.

f MoE values are those reported in Kuiper-Goodman et al. (2010) for the 19 to 30 year age group with no ML and no ADAF.

g MoE values are those reported in Kuiper-Goodman et al. (2010) for the 31 to 50 year age group with no ML and no ADAF.

h MoE values for the 51 to 70 year age group are the same as for the 12 to 18 year age group which was the most conservative of the adult groups from Kuiper-Goodman et al. (2010) with no ML and no ADAF.

l The lifetime MoE is calculated by multiplying the number of years in each age group by the age-specific MoE, summing the resulting totals for all age groups and dividing the grand total by 70 years; the lifetime MoE was calculated by Cantox; other data were from the Kuiper-Goodman et al. (2010) publication.

The revised calculations to consider possible childhood sensitivity indicate that, although the application of the ADAF reduced the lifetime MoE, the resultant value was still greater than 5,000 under all scenarios, and hence, cancer risk is less that 1 in 100,000. Using the relationship of an MoE of 5,000 equating to 1 in 100,000 risk level, the lowest MoE in Table 5 of 6,288 (90th percentile all commodities), would correspond to a risk level of 1 in 125,760, which is essentially negligible.

## Summary

This assessment demonstrates that exposures to OTA from the diets of Canadians are three to four orders of magnitude below doses that have been associated with adverse effects in animals. Also, there is no adequate evidence demonstrating that current dietary exposures to OTA may be associated with adverse effects in humans.

The results of the Kuiper-Goodman et al. (2010) risk assessment, when considered on a lifetime basis indicate that, even in the absence of OTA standards, exposures associated with historical dietary intake would not be associated with a significant risk of adverse health effects. The lifetime MoE up to the 90th percentile based on historical OTA-concentration data for foods considered to be the major sources of dietary exposure does not exceed 5,000 even when early life sensitivity is considered; moreover, there are considerable biological data to support that a safety factor lower than 5,000 could have been used for OTA in determination of proposed MLs.

These biological data supporting a lower safety factor than 5,000 for OTA include the following:

- No evidence from human data that current exposures are associated with adverse effects; recent data support that aristolochic acid and not OTA is the most likely environmental factor in BEN;
- Recent research providing support for many epigenetic or threshold-based mechanisms of action for OTA;
- Rat studies demonstrate a threshold (no response at low doses);
- Although not a classical α-2u-globulin renal carcinogen, some involvement of this protein has been demonstrated which may explain the uncommonly potent response in male rats; α-2u-globulin is not present in humans;
- OTA is negative in most genotoxicity assays including those with highest specificity; a weak response, at most, was noted in others;
- OTA is highly bound in plasma (based on data from one volunteer) with very low levels of free active OTA (greater plasma binding than in the pig);
- The incidence of DNA adducts related to OTA is lower than expected for potent genotoxic carcinogens;
- The dose response results noted for the male rat were determined to have a poor “goodness of fit” when fitted to models used to calculate bench mark doses; the poorest was the multistage model which was the model used to calculate the TD_05_;
- The rat study used to determine the TD_05_ was a gavage study; this route of administration was demonstrated to be associated with higher cancer risk in rats compared to that observed in rats when OTA was administered by the diet which is the route relevant to humans.

Also, DNA repair, apoptosis, immunosurveillance mechanisms, usually discounted in risk assessment, are effective particularly when the frequency of genetic events is low (related to weak potency of OTA as opposed to cellular responses to potent mutagens which would be overwhelming).

This reassessment demonstrates that exposures to OTA from the diets of Canadians are three to four orders of magnitude below doses that have been associated with adverse effects in animals and that there is no adequate evidence demonstrating that current dietary exposures to OTA may be associated with adverse effects in humans. Accordingly, the need for MLs that are currently proposed for OTA in Canada, including infant cereals, has not been demonstrated.

## References

[b1] Adler M, Müller K, Rached E, Dekant W, Mally A (2009). Modulation of key regulators of mitosis linked to chromosomal instability is an early event in ochratoxin A carcinogenicity. Carcinogenesis.

[b2] Ali R, Mittelstaedt RA, Shaddock JG, Bhalli JA, Heflich RH (2010). Micronucleus induction by ochratoxin A in CH9 and TK6 cells. Environ Mol Mutagen.

[b3] Arbillaga L, Azqueta A, van Delft JH, López de Cerain A (2007). *In vitro* gene expression data supporting a DNA non-reactive genotoxic mechanism for ochratoxin A. Toxicol Appl Pharmacol.

[b4] Arlt VM, Stiborová M, vom Brocke J, Simões ML, Lord GM, Nortier JL, Hollstein M, Phillips DH, Schmeiser HH (2007). Aristolochic acid mutagenesis: molecular clues to the aetiology of Balkan endemic nephropathy-associated urothelial cancer. Carcinogenesis.

[b5] Auffray Y, Boutibonnes P (1986). Evaluation of the genotoxic activity of some mycotoxins using Escherichia coli in the SOS spot test. Mutat Res.

[b6] Bansal J, Pantazopoulos P, Tam J, Cavlovic P, Kwong K, Turcotte AM, Lau BP, Scott PM (2011). Surveys of rice sold in Canada for aflatoxins, ochratoxin A and fumonisins. Food Addit Contam Part A Chem Anal Control Expo Risk Assess.

[b7] Bartsch H, Malaveille C, Camus AM, Martel-Planche G, Brun G, Hautefeuille A, Sabadie N, Barbin A, Kuroki T, Drevon C, Piccoli C, Montesano R (1980). Validation and comparative studies on 180 chemicals with S. typhimurium strains and V79 Chinese hamster cells in the presence of various metabolizing systems. Mutat Res.

[b8] Bendele AM, Neal SB, Oberly TJ, Thompson CZ, Bewsey BJ, Hill LE, Rexroat MA, Carlton WW, Probst GS (1985). Evaluation of ochratoxin A for mutagenicity in a battery of bacterial and mammalian cell assays. Food Chem Toxicol.

[b9] Bose S, Sinha SP (1994). Modulation of ochratoxin-produced genotoxicity in mice by vitamin C. Food Chem Toxicol.

[b10] Brambilla G, Martelli A (2004). Failure of the standard battery of short-term tests in detecting some rodent and human genotoxic carcinogens. Toxicology.

[b11] Brusick D (1986). Genotoxic effects in cultured mammalian cells produced by low pH treatment conditions and increased ion concentrations. Environ Mutagen.

[b12] Castegnaro M, Mohr U, Pfohl-Leszkowicz A, Estève J, Steinmann J, Tillmann T, Michelon J, Bartsch H (1998). Sex- and strain-specific induction of renal tumors by ochratoxin A in rats correlates with DNA adduction. Int J Cancer.

[b13] Castegnaro M, Canadas D, Vrabcheva T, Petkova-Bocharova T, Chernozemsky IN, Pfohl-Leszkowicz A (2006). Balkan endemic nephropathy: role of ochratoxins A through biomarkers. Mol Nutr Food Res.

[b14] Cavin C, Delatour T, Marin-Kuan M, Holzhäuser D, Higgins L, Bezençon C, Guignard G, Junod S, Richoz-Payot J, Gremaud E, Hayes JD, Nestler S, Mantle P, Schilter B (2007). Reduction in antioxidant defenses may contribute to ochratoxin A toxicity and carcinogenicity. Toxicol Sci.

[b15] Commission of the European Communities (2006). Commission Regulation (EC) No 1881/2006 19 December 2006 setting maximum levels for certain contaminants in foodstuffs (Text with EEA relevance) [L364]. Off J Eur Union.

[b16] Cooray R (1984). Effects of some mycotoxins on mitogen-induced blastogenesis and SCE frequency in human lymphocytes. Food Chem Toxicol.

[b17] Coronel MB, Sanchis V, Ramos AJ, Marin S (2010). Review. Ochratoxin A: presence in human plasma and intake estimation. Food Sci Technol Int.

[b18] Cosyns JP, Jadoul M, Squifflet JP, De Plaen JF, Ferluga D, van Ypersele de Strihou C (1994). Chinese herbs nephropathy: a clue to Balkan endemic nephropathy?. Kidney Int.

[b19] Creppy EE, Kane A, Dirheimer G, Lafarge-Frayssinet C, Mousset S, Frayssinet C (1985). Genotoxicity of ochratoxin A in mice: DNA single-strand break evaluation in spleen, liver and kidney. Toxicol Lett.

[b20] Czakai K, Müller K, Mosesso P, Pepe G, Schulze M, Gohla A, Patnaik D, Dekant W, Higgins JM, Mally A (2011). Perturbation of mitosis through inhibition of histone acetyltransferases: the key to ochratoxin a toxicity and carcinogenicity?. Toxicol Sci.

[b21] Degen GH, Gerber MM, Obrecht-Pflumio S, Dirheimer G (1997). Induction of micronuclei with ochratoxin A in ovine seminal vesicle cell cultures. Arch Toxicol.

[b22] Delatour T, Mally A, Richoz J, Ozden S, Dekant W, Ihmels H, Otto D, Gasparutto D, Marin-Kuan M, Schilter B, Cavin C (2008). Absence of 2 -deoxyguanosine-carbon 8-bound ochratoxin A adduct in rat kidney DNA monitored by isotope dilution LC-MS/MS. Mol Nutr Food Res.

[b23] Dietrich DR, Heussner AH, O'Brien E (2005). Ochratoxin A: comparative pharmacokinetics and toxicological implications (experimental and domestic animals and humans). Food Addit Contam.

[b24] Dopp E, Müller J, Hahnel C, Schiffmann D (1999). Induction of genotoxic effects and modulation of the intracellular calcium level in Syrian hamster embryo (SHE) fibroblasts caused by ochratoxin A. Food Chem Toxicol.

[b25] Duarte SC, Pena A, Lino CM (2011). Human ochratoxin a biomarkers-from exposure to effect. Crit Rev Toxicol.

[b26] EFSA (2006). Opinion of the Scientific Panel on Contaminants in the Food Chain on a request from the Commission related to ochratoxin A in food (Question number: EFSA-Q-2005-154, adopted on 4 April 2006 by European Food Safety Authority). EFSA J.

[b27] Ehrlich V, Darroudi F, Uhl M, Steinkellner H, Gann M, Majer BJ, Eisenbauer M, Knasmüller S (2002). Genotoxic effects of ochratoxin A in human-derived hepatoma (HepG2) cells. Food Chem Toxicol.

[b28] El Adlouni C, Pinelli E, Azémar B, Zaoui D, Beaune P, Pfohl-Leszkowicz A (2000). Phenobarbital increases DNA adduct and metabolites formed by ochratoxin A: role of CYP 2C9 and microsomal glutathione-S-transferase. Environ Mol Mutagen.

[b29] Faucet V, Pfohl-Leszkowicz A, Dai J, Castegnaro M, Manderville RA (2004). Evidence for covalent DNA adduction by ochratoxin A following chronic exposure to rat and subacute exposure to pig. Chem Res Toxicol.

[b30] Föllmann W, Lucas S (2003). Effects of the mycotoxin ochratoxin A in a bacterial and a mammalian *in vitro* mutagenicity test system. Arch Toxicol.

[b31] Föllmann W, Hillebrand IE, Creppy EE, Bolt HM (1995). Sister chromatid exchange frequency in cultured isolated porcine urinary bladder epithelial cells (PUBEC) treated with ochratoxin A and alpha. Arch Toxicol.

[b32] Galloway SM, Marshall RR, Ishidate M, Brusick D, Ashby J, Myhr BC (1991). Genotoxicity under extreme culture conditions, a report from ICPEMC Task Group 9 (International Commission for Protection Against Environmental Mutagens and Carcinogens). Mutat Res.

[b33] Gautier J, Richoz J, Welti DH, Markovic J, Gremaud E, Guengerich FP, Turesky RJ (2001). Metabolism of ochratoxin A: absence of formation of genotoxic derivatives by human and rat enzymes. Chem Res Toxicol.

[b34] Gluhovschi G, Margineanu F, Velciov S, Gluhovschi C, Bob F, Petrica L, Bozdog G, Trandafirescu V, Modalca M (2011). Fifty years of Balkan endemic nephropathy in Romania: some aspects of the endemic focus in the Mehedinti county. Clin Nephrol.

[b35] Green MH, Muriel WJ (1976). Mutagen testing using TRP+ reversion in Escherichia coli. Mutat Res.

[b36] Grollman AP, Jelakovic B (2007). Role of environmental toxins in endemic (Balkan) nephropathy. October 2006, Zagreb, Croatia. J Am Soc Nephrol.

[b37] Grollman AP, Shibutani S, Moriya M, Miller F, Wu L, Moll U, Suzuki N, Fernandes A, Rosenquist T, Medverec Z, Jakovina K, Brdar B, Slade N, Turesky RJ, Goodenough AK, Rieger R, Vukelic M, Jelakovic B (2007). Aristolochic acid and the etiology of endemic (Balkan) nephropathy. Proc Natl Acad Sci USA.

[b38] Gross-Steinmeyer K, Weymann J, Hege HG, Metzler M (2002). Metabolism and lack of DNA reactivity of the mycotoxin ochratoxin a in cultured rat and human primary hepatocytes. J Agric Food Chem.

[b39] Grosse Y, Baudrimont I, Castegnaro M, Betbeder AM, Creppy EE, Dirheimer G, Pfohl-Leszkowicz A (1995). Formation of ochratoxin A metabolites and DNA-adducts in monkey kidney cells. Chem Biol Interact.

[b40] Hagelberg S, Hult K, Fuchs R (1989). Toxicokinetics of ochratoxin A in several species and its plasma-binding properties. J Appl Toxicol.

[b41] Halmes NC, Roberts SM, Tolson JK, Portier CJ (2000). Reevaluating cancer risk estimates for short-term exposure scenarios. Toxicol Sci.

[b42] Health Canada (2006). Guidelines for Canadian Drinking Water Quality: Guideline Technical Document—Arsenic.

[b43] Health Canada (2009). Information Document on Health Canada's Proposed Maximum Limits (Standards) for the Presence of Mycotoxin Ochratoxin A in Foods: Revised-February 2009.

[b44] Hibi D, Suzuki Y, Ishii Y, Jin M, Watanabe M, Sugita-Konishi Y, Yanai T, Nohmi T, Nishikawa A, Umemura T (2011). Site-specific *in vivo* mutagenicity in the kidney of gpt delta rats given a carcinogenic dose of ochratoxin A. Toxicol Sci.

[b45] IARC (International Agency for Research on Cancer) (1993). Ochratoxin A. http://monographs.iarc.fr/ENG/Monographs/vol56/index.php.

[b46] JECFA (Joint FAO/WHO Expert Committee on Food Additives) (2008). Ochratoxin A (addendum). http://whqlibdoc.who.int/publications/2008/9789241660594_eng.pdf.

[b47] Jennings-Gee JE, Tozlovanu M, Manderville R, Miller MS, Pfohl-Leszkowicz A, Schwartz GG (2010). Ochratoxin A: In Utero Exposure in Mice Induces Adducts in Testicular DNA. Toxins (Basel).

[b48] Kamp HG, Eisenbrand G, Janzowski C, Kiossev J, Latendresse JR, Schlatter J, Turesky RJ (2005). Ochratoxin A induces oxidative DNA damage in liver and kidney after oral dosing to rats. Mol Nutr Food Res.

[b49] Kanki K, Nishikawa A, Masumura K, Umemura T, Imazawa T, Kitamura Y, Nohmi T, Hirose M (2005). *In vivo* mutational analysis of liver DNA in gpt delta transgenic rats treated with the hepatocarcinogens N-nitrosopyrrolidine, 2-amino-3-methylimidazo [4,5-f]quinoline, and di(2-ethylhexyl)phthalate. Mol Carcinog.

[b50] Kirkland D, Aardema M, Henderson L, Müller L (2005). Evaluation of the ability of a battery of three *in vitro* genotoxicity tests to discriminaterodent carcinogens and non-carcinogens I. Sensitivity, specificity and relative predictivity. Mutat Res.

[b51] Klaric MS, Darabos D, Rozgaj R, Kasuba V, Pepeljnjak S (2010). Beauvericin and ochratoxin A genotoxicity evaluated using the alkaline comet assay: single and combined genotoxic action. Arch Toxicol.

[b52] Krivobok S, Olivier P, Marzin DR, Seigle-Murandi F, Steiman R (1987). Study of the genotoxic potential of 17 mycotoxins with the SOS Chromotest. Mutagenesis.

[b53] Krogh P, Axelsen NH, Elling F, Gyrd-Hansen N, Hald B, Hyldgaard-Jensen J, Larsen AE, Madsen A, Mortensen HP, Moller T, Petersen OK, Ravnskov U, Rostgaard M, Aalund O (1974). Experimental porcine nephropathy. Changes of renal function and structure induced by ochratoxin A- contaminated feed. Acta Pathol Microbiol Scand Suppl.

[b54] Krogh P, Elling F, Friis C, Hald B, Larsen AE, Lillehøj EB, Madsen A, Mortensen HP, Rasmussen F, Ravnskov U (1979). Porcine nephropathy induced by long-term ingestion of ochratoxin A. Vet Pathol.

[b55] Kuiper-Goodman T, Magan N, Olsen M (2004). Risk assessment and risk management of mycotoxins in food. Mycotoxins in Food: Detection and Control.

[b56] Kuiper-Goodman T, Scott PM (1989). Risk assessment of the mycotoxin ochratoxin A. Biomed Environ Sci.

[b57] Kuiper-Goodman T, Hilts C, Billiard SM, Kiparissis Y, Richard ID, Hayward S (2010). Health risk assessment of ochratoxin A for all age-sex strata in a market economy. Food Addit Contam Part A Chem Anal Control Expo Risk Assess.

[b58] Kumari D, Sinha SP (1994). Effect of retinol on ochratoxin-produced genotoxicity in mice. Food Chem Toxicol.

[b59] Lebrun S, Golka K, Schulze H, Föllmann W (2006). Glutathione S-transferase polymorphisms and ochratoxin A toxicity in primary human urothelial cells. Toxicology.

[b60] Li J, Yin S, Dong Y, Fan L, Hu H (2011). p53 activation inhibits ochratoxin A-induced apoptosis in monkey and human kidney epithelial cells via suppression of JNK activation. Biochem Biophys Res Commun.

[b61] Malaveile C, Brun G, Bartsch H, Castegnaro M, Plestina R, Dirheimer G, Chernozemsky IN, Bartsch H (1991). Genotoxicity of ochratoxin A and structurally related compounds in Escheriehia coli strains: studies on their mode of action. Mycotoxins, EndemicNephropathy and Urinary Tract Tumours. IARC Scientific Publications no 115.

[b62] Mally A, Dekant W (2009). Mycotoxins and the kidney: modes of action for renal tumor formation by ochratoxin A in rodents. Mol Nutr Food Res.

[b63] Mally A, Zepnik H, Wanek P, Eder E, Dingley K, Ihmels H, Völkel W, Dekant W (2004). Ochratoxin A: lack of formation of covalent DNA adducts. Chem Res Toxicol.

[b64] Mally A, Pepe G, Ravoori S, Fiore M, Gupta RC, Dekant W, Mosesso P (2005a). Ochratoxin a causes DNA damage and cytogenetic effects but no DNA adducts in rats. Chem Res Toxicol.

[b65] Mally A, Völkel W, Amberg A, Kurz M, Wanek P, Eder E, Hard G, Dekant W (2005b). Functional, biochemical, and pathological effects of repeated oral administration of ochratoxin A to rats. Chem Res Toxicol.

[b66] Mally A, Hard GC, Dekant W (2007). Ochratoxin A as a potential etiologic factor in endemic nephropathy: lessons from toxicity studies in rats. Food Chem Toxicol.

[b67] Manderville RA (2005). A case for the genotoxicity of ochratoxin A by bioactivation and covalent DNA adduction. Chem Res Toxicol.

[b68] Manolova Y, Manolov G, Parvanova L, Petkova-Bocharova T, Castegnaro M, Chernozemsky IN (1990). Induction of characteristic chromosomal aberrations, particularly X-trisomy, in cultured human lymphocytes treated by ochratoxin A, a mycotoxin implicated in Balkan endemic nephropathy. Mutat Res.

[b69] Mantle PG (2009). Minimum tolerable exposure period and maximum threshold dietary intake of ochratoxin A for causing renal cancer in male Dark Agouti rats. Food Chem Toxicol.

[b70] Mantle P, Kulinskaya E (2010). Lifetime, low-dose ochratoxin A dietary study on renal carcinogenesis in male Fischer rats. Food Addit Contam Part A Chem Anal Control Expo Risk Assess.

[b71] Mantle PG, Nagy JM (2008). Binding of ochratoxin A to a urinary globulin: a new concept to account for gender difference in rat nephrocarcinogenic responses. Int J Mol Sci.

[b72] Mantle P, Kulinskaya E, Nestler S (2005). Renal tumourigenesis in male rats in response to chronic dietary ochratoxin A. Food Addit Contam.

[b73] Mantle PG, Faucet-Marquis V, Manderville RA, Squillaci B, Pfohl-Leszkowicz A (2010). Structures of covalent adducts between DNA and ochratoxin a: a new factor in debate about genotoxicity and human risk assessment. Chem Res Toxicol.

[b74] Marin-Kuan M, Nestler S, Verguet C, Bezençon C, Piguet D, Mansourian R, Holzwarth J, Grigorov M, Delatour T, Mantle P, Cavin C, Schilter B (2006). A toxicogenomics approach to identify new plausible epigenetic mechanisms of ochratoxin a carcinogenicity in rat. Toxicol Sci.

[b75] Marin-Kuan M, Cavin C, Delatour T, Schilter B (2008). Ochratoxin A carcinogenicity involves a complex network of epigenetic mechanisms. Toxicon.

[b76] Miljkovic A, Pfohl-Leszkowicz A, Dobrota M, Mantle PG (2003). Comparative responses to mode of oral administration and dose of ochratoxin A or nephrotoxic extract of Penicillium polonicum in rats. Exp Toxicol Pathol.

[b77] Mori H, Kawai K, Ohbayashi F, Kuniyasu T, Yamazaki M, Hamasaki T, Williams GM (1984). Genotoxicity of a variety of mycotoxins in the hepatocyte primary culture/DNA repair test using rat and mouse hepatocytes. Cancer Res.

[b78] Mosesso P, Cinelli S, Piñero J, Bellacima R, Pepe G (2008). *In vitro* cytogenetic results supporting a DNA nonreactive mechanism for ochratoxin A, potentially relevant for its carcinogenicity. Chem Res Toxicol.

[b79] Murdoch DJ, Krewski D, Wargo J (1992). Cancer risk assessment with intermittent exposure. Risk Anal.

[b80] Nedelko T, Arlt VM, Phillips DH, Hollstein M (2009). TP53 mutation signature supports involvement of aristolochic acid in the aetiology of endemic nephropathy-associated tumours. Int J Cancer.

[b81] Nestmann ER, Bryant DW, Carr CJ (1996). Toxicological significance of DNA adducts: summary of discussions with an expert panel. Regul Toxicol Pharmacol.

[b82] NTP (National Toxicology Program) (1989). Toxicology and Carcinogenesis Studies of Ochratoxin A (CAS No. 303-47-9) in F344/N rats (Gavage Studies). Technical Report Series no 358. Department of Health and Human Services.

[b83] NTP (National Toxicology Program) (2011). Aristolochic acids. Rep Carcinog.

[b84] O'Brien E, Heussner AH, Dietrich DR (2001). Species-, sex-, and cell type-specific effects of ochratoxin A and B. Toxicol Sci.

[b85] Palma N, Cinelli S, Sapora O, Wilson SH, Dogliotti E (2007). Ochratoxin A-induced mutagenesis in mammalian cells is consistent with the production of oxidative stress. Chem Res Toxicol.

[b86] Pfohl-Leszkowicz A (2009). Ochratoxin A and aristolochic acid involvement in nephropathies and associated urothelial tract tumours. Arh Hig Rada Toksikol.

[b87] Pfohl-Leszkowicz A, Castegnaro M (2005). Further arguments in favour of direct covalent binding of Ochratoxin A (OTA) after metabolic biotransformation. Food Addit Contam.

[b88] Pfohl-Leszkowicz A, Chakor K, Creppy E, Dirheimer G, Castegnaro M, Plestina R, Dirheimer G, Chernozemsky IN, Bartsch H (1991). DNA adduct formation in mice treated with ochratoxin A. Mycotoxins, Endemic Nephropathy and Urinary Tract Tumours. (IARC Scientific Publications no 115.

[b89] Pfohl-Leszkowicz A, Grosse Y, Castegnaro M, Nicolov IG, Chernozemsky IN, Bartsch H, Betbeder AM, Creppy EE, Dirheimer G, Phillips DH, Castegnaro M, Bartsch H (1993). Ochratoxin A-related DNA adducts in urinary tract tumours of Bulgarian subjects. Postlabelling Methods for the Detection of DNA Adducts. IARC Scientific Publications no 124.

[b90] Rached E, Pfeiffer E, Dekant W, Mally A (2006). Ochratoxin A: apoptosis and aberrant exit from mitosis due to perturbation of microtubule dynamics?. Toxicol Sci.

[b91] Ringot D, Chango A, Rai M, Varma A (2010). Risk assessment of ochratoxin A (OTA). Mycotoxins in Food, Feed and Bioweapons.

[b92] Schilter B, Marin-Kuan M, Delatour T, Nestler S, Mantle P, Cavin C (2005). Ochratoxin A: potential epigenetic mechanisms of toxicity and carcinogenicity. Food Addit Contam.

[b93] Schlatter C, Studer-Rohr J, Rásonyi T (1996). Carcinogenicity and kinetic aspects of ochratoxin A. Food Addit Contam.

[b94] Simarro Doorten Y, Nijmeijer S, de Nijs-Tjon L, Fink-Gremmels J (2006). Metabolism-mediated Ochratoxin A genotoxicity in the single-cell gel electrophoresis (Comet) assay. Food Chem Toxicol.

[b95] Slade N, Moll UM, Brdar B, Zoric A, Jelakovic B (2009). p53 mutations as fingerprints for aristolochic acid: an environmental carcinogen in endemic (Balkan) nephropathy. Mutat Res.

[b96] Stachurska A, Kozakowska M, Jozkowicz A, Dulak J, Loboda A (2011). Aristolochic acid I and ochratoxin A differentially regulate VEGF expression in porcine kidney epithelial cells–the involvement of SP-1 and HIFs transcription factors. Toxicol Lett.

[b97] Stefanovic V, Polenakovic M (2009). Fifty years of research in Balkan endemic nephropathy: where are we now?. Nephron Clin Pract.

[b98] Stefanovic V, Polenakovic M, Toncheva D (2011). Urothelial carcinoma associated with Balkan endemic nephropath. A worldwide disease. Pathol Biol.

[b99] Stetina R, Votava M (1986). Induction of DNA single-strand breaks and DNA synthesis inhibition by patulin, ochratoxin A, citrinin, and aflatoxin B1 in cell lines CHO and AWRF. Folia Biol (Praha).

[b100] Tam J, Pantazopoulos P, Scott PM, Moisey J, Dabeka RW, Richard ID (2011). Application of isotope dilution mass spectrometry: determination of ochratoxin A in the Canadian Total Diet Study. Food Addit Contam Part A Chem Anal Control Expo Risk Assess.

[b101] Turesky RJ (2005). Perspective: ochratoxin A is not a genotoxic carcinogen. Chem Res Toxicol.

[b102] USDA (US Department of Agriculture) (2000).

[b103] US EPA (US Environmental Protection Agency) (2005a). Guidelines for Carcinogen Risk Assessment. EPA/630/P-03/001B.

[b104] US EPA (US Environmental Protection Agency) (2005b). Supplemental Guidance for Assessing Susceptibility from Early-Life Exposure to Carcinogens. EPA/630/R-03/003F.

[b105] Vettorazzi A, de Tróconiz IF, González-Penas E, Arbillaga L, Corcuera LA, Gil AG, de Cerain AL (2011). Kidney and liver distribution of ochratoxin A in male and female F344 rats. Food Chem Toxicol.

[b106] Waddell WJ (2006). Critique of dose response in carcinogenesis. Hum Exp Toxicol.

[b107] Wehner FC, Thiel PG, van Rensburg SJ, Demasius IP (1978). Mutagenicity to Salmonella typhimurium of some Aspergillus and Penicillium mycotoxins. Mutat Res.

[b108] Wu F (2004). Mycotoxin risk assessment for the purpose of setting international regulatory standards. Environ Sci Technol.

[b109] Wu Q, Dohnal V, Huang L, Kuca K, Wang X, Chen G, Yuan Z (2011). Metabolic pathways of ochratoxin A. Curr Drug Metab.

[b110] Würgler FE, Friederich U, Schlatter J (1991). Lack of mutagenicity of ochratoxin A and B, citrinin, patulin and cnestine in Salmonella typhimurium TA102. Mutat Res.

[b111] Zeiger E, Anderson B, Haworth S, Lawlor T, Mortelmans K (1988). Salmonella mutagenicity tests: IV.Results from the testing of 300 chemicals. Environ Mol Mutagen.

[b112] Zeljezic D, Domijan AM, Peraica M (2006). DNA damage by ochratoxin A in rat kidney assessed by the alkaline comet assay. Braz J Med Biol Res.

[b113] Zepnik H, Pähler A, Schauer U, Dekant W (2001). Ochratoxin A-induced tumor formation: is there a role of reactive ochratoxin A metabolites?. Toxicol Sci.

[b114] Zepnik H, Völkel W, Dekant W (2003). Toxicokinetics of the mycotoxin ochratoxin A in F 344 rats after oral administration. Toxicol Appl Pharmacol.

[b115] Zhang X, Boesch-Saadatmandi C, Lou Y, Wolffram S, Huebbe P, Rimbach G (2009). Ochratoxin A induces apoptosis in neuronal cells. Genes Nutr.

